# The BET inhibitor attenuates the inflammatory response and cell migration in human microglial HMC3 cell line

**DOI:** 10.1038/s41598-021-87828-1

**Published:** 2021-04-23

**Authors:** Mina Baek, Eunyoung Yoo, Hae In Choi, Ga Yeong An, Jin Choul Chai, Young Seek Lee, Kyoung Hwa Jung, Young Gyu Chai

**Affiliations:** 1 Department of Molecular and Life Science, Hanyang University, Ansan, 15588 Republic of Korea; 2 Institute of Natural Science and Technology, Hanyang University, Ansan, 15588 Republic of Korea; 3 Department of Bionanotechnology, Hanyang University, Seoul, 04673 Republic of Korea; 4 College of Veterinary Medicine, Seoul National University, Seoul, 08826 Republic of Korea; 5 Department of Biopharmaceutical System, Gwangmyeong Convergence Technology Campus of Korea Polytechnic II, Gwangmyeong, 14222 Republic of Korea

**Keywords:** Neuroimmunology, Gene expression

## Abstract

Microglia, resident macrophages of the brain that act as primary immune cells, play essential roles in innate immunity and neuroinflammatory pathologies. Microglial cells are rapidly activated in response to infection and inflammation/injury, associated with the expression of proinflammatory genes and secretion of cytokines. The bromodomain and extra-terminal (BET) inhibitor JQ1 has been shown to be an epigenetic agent that reduces inflammation. In this study, we investigated the mechanisms underlying the anti-inflammatory and anti-migratory functions of JQ1 and the genes targeted by JQ1 in lipopolysaccharide (LPS)-activated human microglial clone 3 (HMC3) cells using RNA-sequencing (RNA-seq). We analyzed the pattern of inflammation-related genes (chemokines, cytokines, and interferon-stimulated genes) and migration-related genes with JQ1 treatment from differentially expressed genes analysis in HMC3 cells. We found that LPS-induced IRF1 directly regulated inflammation- and migration-related genes and that JQ1 significantly reduced IRF1 and its target genes. Additionally, IRF1 attenuation significantly downregulated target genes and inhibited microglial migration. Our data suggest that the BET inhibitor JQ1 can modulate the inflammatory response and migration through the regulation of LPS-induced IRF1 in human microglia.

## Introduction

Microglia are brain-resident macrophages that regulate brain development, the maintenance of neuronal networks, and injury repair^1,2^. Furthermore, microglia continuously survey the central nervous system microenvironment and maintain homeostasis under normal physiological conditions^3^. Microglia also play critical roles in the innate immune system and neuroinflammation in the brain. In response to injury or inflammatory stimuli, microglial cells are rapidly activated and promote neuroinflammatory processes through the secretion of various chemokines and cytokines. Microglia act as neuromodulators that can modify the morphology and phagocytic activity^4,5^. These regulatory effects of microglial cells can affect neurons by modulating synapse formation and neuronal survival^6^.

Lipopolysaccharide (LPS) functions as a potent activator of the immune system^7^. LPS induces microglial activation, produces proinflammatory chemokines and cytokines, and elicits a wide range of effects on cell adhesion, migration, survival, and cell death^8,9^. In vitro treatment of microglial cells with proinflammatory stimuli such as LPS and interferon gamma (IFNγ) induces M1 polarization characterized by the increased expression of proinflammatory molecules, such as IL1β, IFNγ, CXCL10, NOS, and COX2^10^.

Microglial cells activated by the inflammatory response migrate to the affected site and release various proinflammatory molecules. Therefore, increased migration may also be a feature of activated microglial cells^3,11,12^.

Bromodomains are served as regulators of protein–protein interactions in different cellular processes such as transcription and chromatin remodeling^13^. The bromodomain and extra-terminal (BET) family proteins bind acetylated histones in the enhancer or promoter regions of inflammatory genes. The BET family proteins comprise bromodomain-containing protein 2 (BRD2), BRD3, BRD4, and BRDT, which are epigenetic reader proteins^14^. In particular, BRD4 acts in the key steps of transcription factors (TFs) recruitment, enhancer and mediator complex assembly, and transcriptional elongation of oncogenes and proinflammatory chemokines or cytokines^15^. Furthermore, many in vitro studies have found that BET family proteins regulate the transcription of proinflammatory genes^16^.

JQ1 is a selective inhibitor of BET proteins that competitively binds acetylated lysine residues on chromatin^17^. BRD4 inhibition by JQ1 was demonstrated to attenuate LPS‐induced expression of proinflammatory cytokines and suppress inflammatory reactions in HAPI murine microglial cells^18^. In our previous studies, we reported that BET inhibitor JQ1 significantly reduces critical inflammatory genes in LPS-stimulated BV-2 murine microglial cells^19^ and murine primary bone marrow-derived macrophages^20^, implying that the BET proteins control to neuroinflammation.

In the present study, we performed RNA-seq for gene expression profiling of LPS + JQ1-treated human microglial clone 3 (HMC3) cells compared with LPS-treated HMC3 cells. Our results show that the BET inhibitor JQ1 attenuated LPS-induced inflammatory and migratory responses, revealing the regulatory effects of JQ1 in microglia activation. Additionally, JQ1 inhibited the LPS-induced IRF1, which suppressed the migration of microglia by regulating migration-related genes. This manuscript describes the first study to performed transcriptome analyses of neuroinflammation in human microglial cell lines using RNA-seq analysis to the best of our knowledge. We also showed an anti-inflammatory and anti-migratory effect for JQ1 on genome-wide mRNA level changes. These results provide a better understanding of the molecular signatures of neuroinflammation in human microglial cell lines. It also provides the basis for BET proteins to play an essential role in regulating inflammation and migration genes.

## Results

### Analysis of cell morphology and viability of LPS- or JQ1-treated HMC3 cells

**Figure 1:**
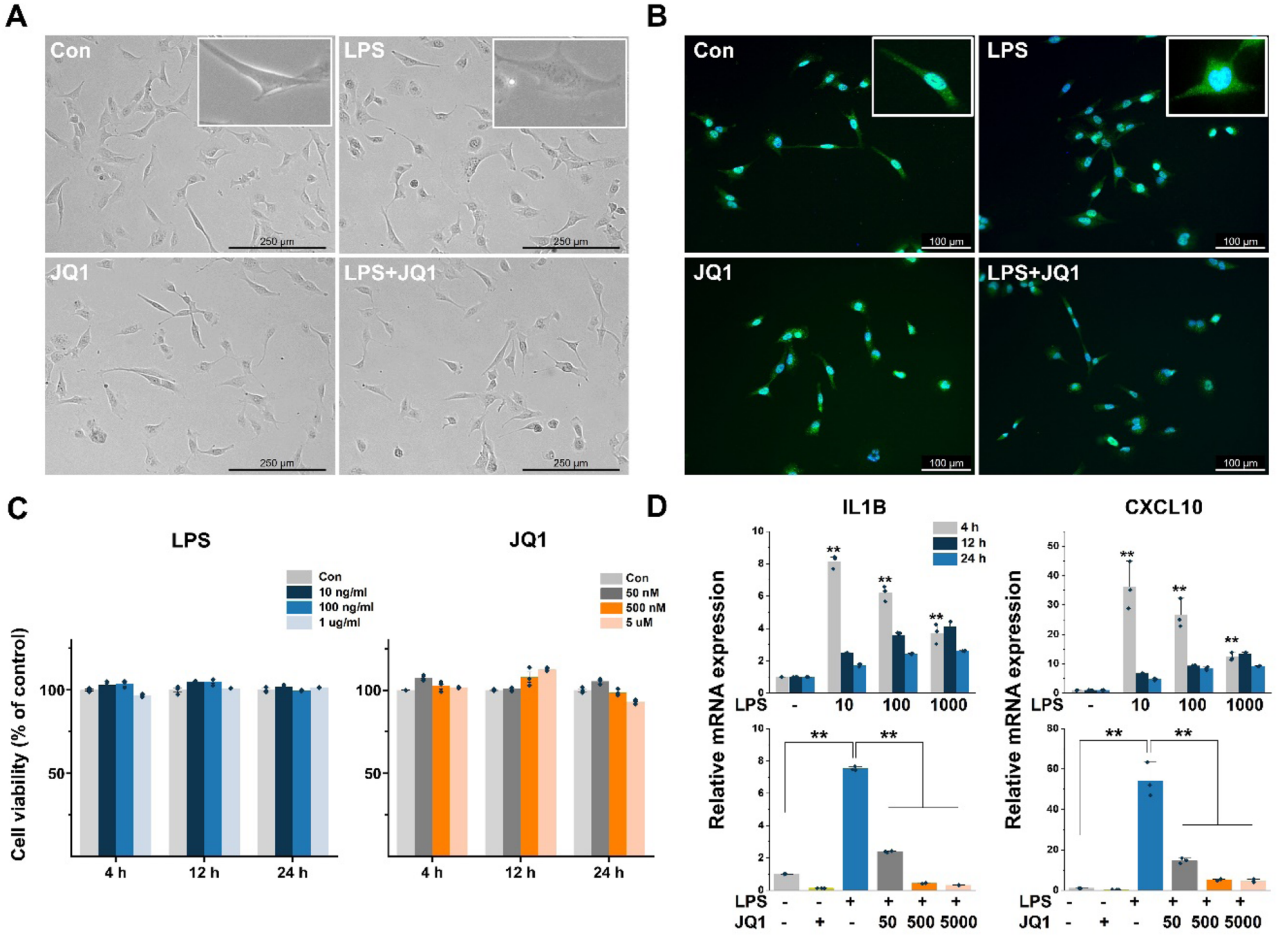
Effects of LPS and JQ1 on HMC3 cells and the expression of inflammatory response-related genes in LPS- and JQ1-treated cells. (**A**) Morphology of HMC3 cells after treatment with LPS, JQ1, and LPS + JQ1 for 4 h. (**B**) HMC3 cells were cultured for 4 h after treatment with LPS (100 ng/ml) and JQ1 (500 nM) and stained for IBA1 (green fluorescence), a microglia-lineage marker. Nuclei were counterstained with DAPI (blue). (**C**) HMC3 cells were treated with LPS and JQ1 at different concentrations for different durations (4 h, 24 h, and 48 h). The viability of the HMC3 cells was determined using the WST-1 assay. (**D**) HMC3 cells were treated with LPS at different doses (10, 100, and 1000 ng/ml) for 4 h. Inflammatory genes were significantly upregulated in cells treated with LPS compared to DMSO-treated control cells (upper panel). HMC3 cells were treated with LPS (100 ng/ml) and JQ1 at different doses (50, 500, and 5000 nM) for 4 h. Inflammatory genes were significantly downregulated in cells treated with JQ1 (bottom panel). Gene expression was normalized to GAPDH transcript levels. The data represent three independent experiments. The values are the mean ± SEM of triplicate experiments (*p < 0.05 and **p < 0.001).

In general, microglia of different phenotypes are classified by their morphological features. 'Resting' microglia are characterized as highly ramified, and 'activated' microglia have transformed to an amoeboid phenotype^21^. DMSO- and JQ1-treated HMC3 cells (resting cells) showed a morphology typical of microglia with small cell bodies and multiple, branched, and extensive ramifications of the distal processes. The LPS-treated HMC3 cells (activated cells) tended to exhibit an appearance characteristic of activated microglia that generally had larger cell bodies, fewer branches, and an amoeboid phenotype compared with the DMSO- and JQ1-treated cells (Fig. 1A). Also, some LPS + JQ1-treated HMC3 cells showed an amoeboid phenotype. To validate the HMC3 cells, we performed immunostaining for IBA1, a microglia-lineage marker at 4 h (Fig. 1B) and 24 h (Fig. S1A). The HMC3 cells were strongly expressed IBA1 in both resting and activated microglia. Also, the mRNA expression of microglia-lineage markers (P2RY12 and CSF1R) was confirmed (Fig. S1B).

The effects of LPS and JQ1 on the viability of HMC3 cells were evaluated using the WST-1 assay. The LPS- and JQ1-treated cells were shown no significant differences compared to control cells, suggesting that those cells were not affected by the cellular cytotoxic in our experiments (Fig. 1C).

### Gene induction patterns in HMC3 cells following LPS and LPS + JQ1 treatment

To determine the optimal time and concentration for gene induction by LPS, we analyzed the expression of inflammatory genes in LPS-treated cells compared to control cells. The HMC3 cells were treated with LPS at different doses (10–1000 ng/ml) and at different time points (4 h, 12 h, and 24 h). As shown in Figs. 1D and S1C, the LPS-treated cells were significantly upregulated expressions of the inflammatory genes such as CCL2, CXCL10, IFNB1, IL1B, and TNFa, which peaked at 4 h. Gene expressions of IFNB1 and TNFα were increased slightly further at 10 ng/ml, but no significant difference from 100 ng/ml. Rather, gene expressions of CCL2, IFNB1, and IL1B were increased at 12 h with 100 ng/ml than 10 ng/ml LPS. Next, we examined whether JQ1 could alter the expression of inflammatory genes after an induced inflammatory response with 100 ng/ml LPS. JQ1 significantly suppressed LPS-induced inflammatory genes in a dose-dependent manner (Figs. 1D and S1C). We thus determined the optimal experimental conditions for subsequent transcriptional profiling.

### Differentially expressed genes in LPS-treated HMC3 cells

**Figure 2:**
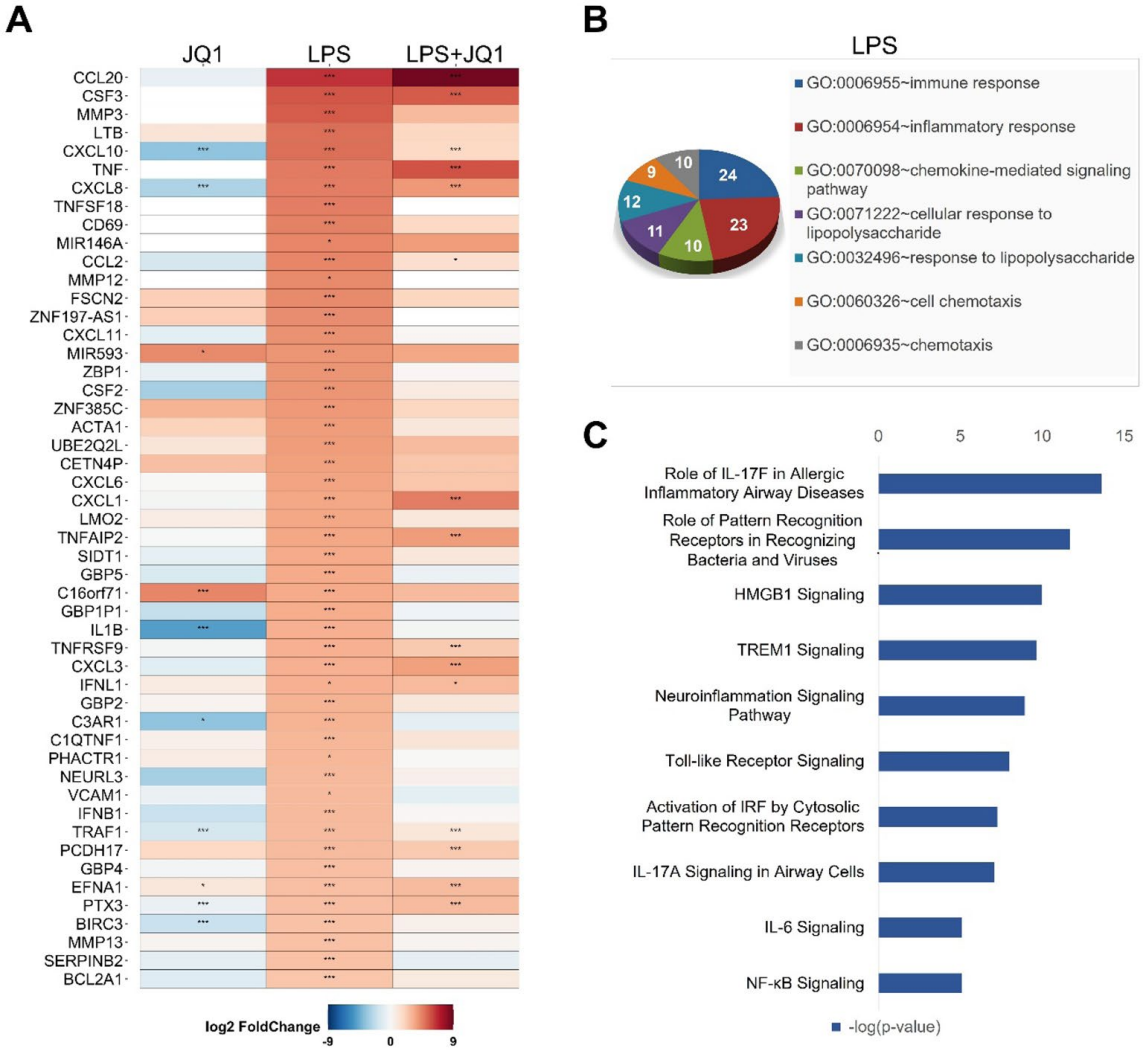
Differentially expressed genes in LPS- and LPS + JQ1-treated HMC3 cells. (**A**) Heat map showing the top 50 upregulated genes in HMC3 cells treated with LPS and LPS + JQ1 for 4 h determined by RNA-seq (*p* ≤ 0.05 and fold change ≥ 1.5 log2). Each experiment was performed in experimental triplicates (n = 3) for each condition, and the results were individually combined. The color scale shown in the heat map represents the log_2_ fold change values. Red cells indicate upregulated gene, while blue cells indicate downregulated genes. The p-value with an asterisk attached in the cell represents *p < 0.05, **p < 0.01, and ***p < 0.001. (**B**) GO term analysis of the biological processes associated with up- and downregulated genes after LPS (100 ng/ml) and JQ1 (500 nM) treatment for 4 h. GO analysis of the number of genes shown in the van diagram. (**C**) Biological pathway analysis of DEGs in cells treated with LPS and LPS + JQ1 using IPA (QIAGEN Inc., https://www.qiagenbioinformatics.com/products/ingenuitypathway-analysis) showed up- and downregulated genes.

To identify the gene signatures in activated microglial cells, we treated HMC3 cells with LPS (100 ng/ml) before cDNA library construction for RNA-seq experiments. Comprehensive transcriptomic analysis revealed that 132 genes were differentially regulated in LPS-treated HMC3 cells. Among these genes, 112 genes were upregulated, whereas 20 genes were downregulated after LPS treatment (*p* ≤ 0.05 and a fold change ≥ 1.5 log_2_). The heat map in Fig. 2A shows the gene expression patterns of the top 50 genes upregulated by LPS. Most of the upregulated genes were inflammatory response- and immune response-related genes, such as CCL20, CSF3, MMP3, LTB, and CXCL10. Next, we performed functional classification analysis of the DEGs using DAVID and classified the results into biological process categories for GO term analysis. The GO term analysis used up- and downregulated genes after treatment with LPS (100 ng/ml) for 4 h. The up- and downregulated genes are listed in Tables S1 and S2, respectively. GO analysis showed that the biological functions of DEGs in the LPS-treated groups focused on the inflammatory response, the response to stimuli, and cell chemotaxis (Fig. 2B). Besides, to understand of the molecular functions of the DEGs, we performed IPA to identify biological pathways. Biological pathway analysis of the transcriptome profiling data revealed the significant enrichment of LPS-mediated pathways. Those pathways are related to the DEGs' immune responses, including genes involved in the role of IL-17F in allergic inflammatory airway disease, the role of pattern recognition receptors in recognizing bacteria and viruses, the neuroinflammation signaling pathway, IL6 signaling, and NF-κB signaling. Other notable pathways included high mobility group box 1 (HMGB1) signaling, triggering receptor expressed on myeloid Cells 1 (TREM1) signaling, Toll-like receptor signaling, and the activation of IRF by cytosolic pattern recognition receptors (Fig. 2C).

### Differentially expressed genes in LPS + JQ1-treated HMC3 cells

To analyze the transcriptome of JQ1-treated activated microglial cells, we performed RNA-seq experiments using a cDNA library of LPS + JQ1-treated HMC3 cells. RNA-seq analysis revealed 556 DEGs in the LPS + JQ1-treated HMC3 cells. Among these genes, 337 genes were upregulated, whereas 237 genes were downregulated in the LPS + JQ1-treated cells compared to control cells (*p* ≤ 0.05 and a fold change ≥ 1.5 log_2_). The heat map in Fig. S2A shows the gene expression patterns of the top 50 genes upregulated in LPS + JQ1-treated cells. Most of the upregulated genes were inflammatory response-related genes, including CCL2, PCDH1, TNF, CSF3, and NKD1. The GO term analysis used up- and downregulated genes after treatment with LPS (100 ng/ml) and JQ1 (500 nM) for 4 h. The up- and downregulated genes are listed in Tables S3 and S4, respectively. The results of GO analysis using DAVID showed that the biological functions of the DEGs in LPS + JQ1-treated cells were related to the inflammatory response and cell–cell signaling and cell migration (Fig. S2B). Biological pathway analysis of the DEGs in LPS + JQ1-treated cells using IPA showed their involvement in IL17A signaling, HMGB1 signaling, NF-κB signaling, IL6 signaling, and neuroinflammation signaling pathways, which were the same pathways indicated by biological pathway analysis of the DEGs in LPS-treated cells. However, some pathways, such as the production of nitric oxide and reactive oxygen species in macrophages, cardiac hypertrophy signaling, CD27 signaling, and IL8 signaling, were enriched LPS + JQ1-treated cells (Fig. S2C).

### Differentially expressed genes in JQ1-treated HMC3 cells

We also evaluated the effect of JQ1 alone on HMC3 cells. A total of 273 genes were upregulated, and 249 genes were downregulated in JQ1-treated cells (Fig. S3A). The GO term analysis used up- and downregulated genes after treatment with JQ1 (500 nM) for 4 h. The up- and downregulated genes are listed in Tables S5 and S6, respectively. Among these genes, several genes are involved in the inflammatory response (CXCL8, CXCL10, IFIT5, IFNLR1, IL1B, IL6, IL15, TNFAIP3, TNFSF4, TNFSF9, and TNFSF10), but most of the DEGs are not. Interestingly, the inflammatory response genes described above were downregulated by JQ1. GO analysis showed that the DEGs' biological functions are associated with the regulation of cell proliferation and the response to LPS (Fig. S3B). Biological pathway analysis of the DEGs in JQ1-treated cells using IPA showed their involvement in HMGB1 signaling, which were the same pathways in the DEGs in LPS- and LPS + JQ1-treated cells. However, some pathways, such as the role of macrophages, fibroblasts, and endothelial cells in rheumatoid arthritis, axonal guidance signaling, ceramide signaling, and STAT3 pathway, were enriched in JQ1-treated cells (Fig. S3C).

### Gene signatures involved in the effect of JQ1 on the LPS-induced inflammatory response

**Figure 3:**
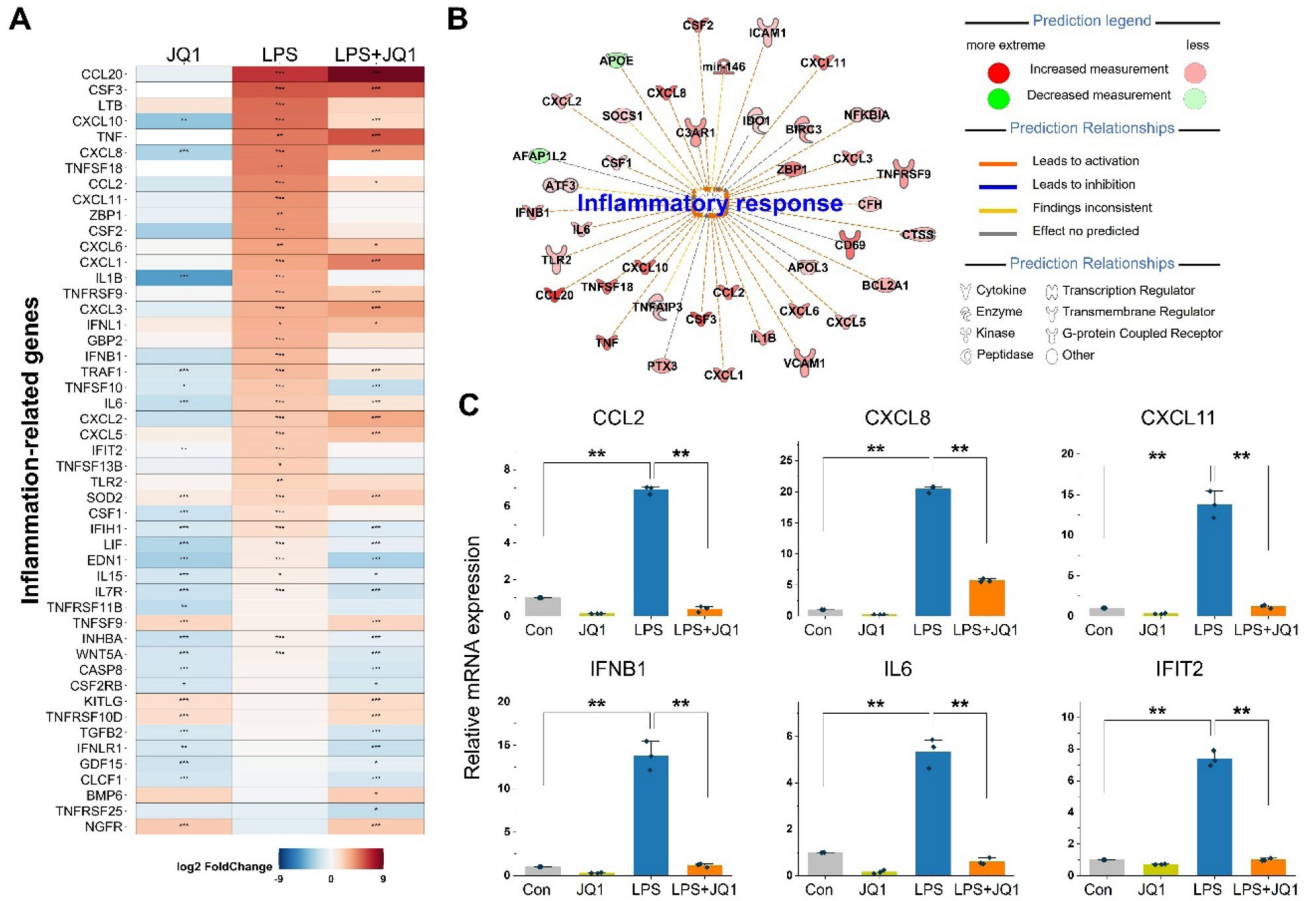
JQ1 inhibits a specific subset of LPS-inducible inflammatory genes. (**A**) Heat map showing chemokine/cytokine and interferon response gene expression in HMC3 cells treated with LPS (100 ng/ml) and JQ1 (500 nM) for 4 h. The color scale shown in the heat map represents the log_2_ fold change values. (**B**) IPA downstream effects analysis presenting the mapping of most significant genes related to inflammatory response genes in LPS-treated cells. The DEGs are colored by predicted activation state, such as activated (red) or suppressed (green). The edges connecting the nodes are colored orange (leading to activation of the downstream node), blue (leading to inhibition), and yellow (if the findings underlying the relationship are inconsistent with the state of the downstream node). (**C**) Expression levels of inflammation-related genes were analyzed by qRT-PCR and normalized to GAPDH transcript levels. Overall, LPS-induced inflammatory genes were inhibited by JQ1. The data represent three independent experiments. The values are the mean ± SEM of triplicate experiments (**p < 0.001).

To investigate whether JQ1 acts as an anti-inflammatory agent in human microglial cells, we exposed HMC3 cells to both LPS and JQ1 and compared their transcriptomic profile from that of the group treated with LPS alone. Most inflammatory genes were upregulated in the activated cells (treated with only LPS) but suppressed in activated HMC3 cells upon JQ1 treatment; these genes include chemokines and cytokines (CCL2, CCL20, CXCL1, CXCL2, CXCL3, CXCL5, CXCL6, CXCL8, CXCL10, and CXCL11), interferon-related genes (IFNB1, IFNL1, IFNRL1, IFIH1, IFIT2, and ZBP1), interleukins and related genes (IL1B, IL6, IL7R, and IL15), and TNFα-related genes (TNF, TNFRSF9, TNFRSF10D, TNFRSF11B, TNFSF10, TNFSF13B, and TNFSF18) (Fig. 3A, C). IPA downstream effects analysis identifies biological processes and functions related to inflammatory responses and predicts whether these processes increase or decrease. Using IPA downstream effects analysis, we found that 39 DEGs, including chemokines/cytokines genes in LPS-treated cells (Fig. 3B). Values indicating the log_2_ fold change and *p*-value in the expression of inflammatory genes in LPS- and LPS + JQ1-treated cells are listed in Table S7. To validate the RNA-seq results, we confirmed the expression of the DEGs by qRT-PCR. Because JQ1 acts as an anti-inflammatory agent, we selected chemokines (CCL2, CXCL8, and CXCL11), cytokines (IFNB1 and IL6), and an interferon response gene (IFIT2) for qRT-PCR experiments (Fig. 3C). The mRNA levels of CCL2, CXCL8, CXCL11, IFNB1, IL6, and IFIT2 were upregulated in LPS-treated HMC3 cells but suppressed in LPS + JQ1-treated HMC3 cells. Overall, these findings confirmed that JQ1 significantly inhibited inflammatory genes induced by LPS.

### Effect of JQ1 on genes involved in the LPS-induced migratory response

**Figure 4:**
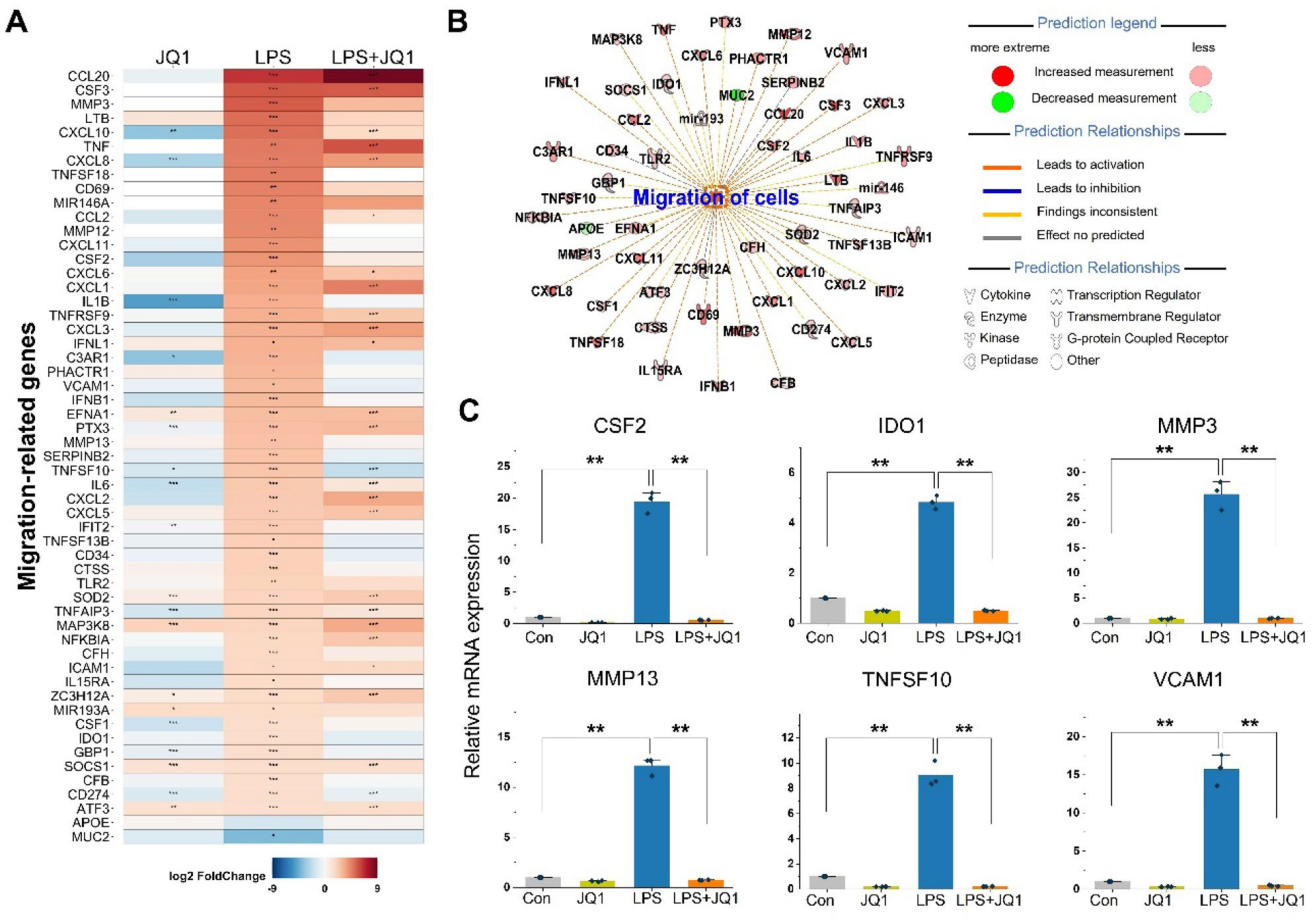
JQ1 inhibits a specific subset of LPS-inducible migratory genes. (**A**) Heat map showing migration-related gene expression in cells treated with LPS (100 ng/ml) and JQ1 (500 nM) at 4 h. The color scale shown in the heat map represents the log_2_ fold change values. (**B**) IPA downstream effects analysis presenting the mapping of most significant genes related to migratory response genes in LPS-treated cells. (**C**) Expression levels of migration-related genes were analyzed by qRT-PCR and normalized to GAPDH transcript levels. Overall, LPS-induced migratory genes were inhibited by JQ1. The data represent three independent experiments. The values are the mean ± SEM of triplicate experiments (**p < 0.001).

The migration of microglial cells is one of the hallmarks of the inflammatory response to the site of injury or inflammation. Inflammation-related genes and migration-related genes were affected by LPS and were also downregulated by JQ1 treatment (Fig. 4A). We found that most of the genes were related to inflammation or migration coincide with chemotaxis related genes (CCL2, CCL20, CXCL1, CXCL2, CXCL5, CXCL8, CXCL10, CSF1, CSF2, ICAM1, IFNB1, IL1D, IL6, TNF, TNFSF18, and VCAM1). Using IPA downstream effects analysis, we found that 56 DEGs in the LPS-treated cells were related to the migratory response (Fig. 4B). Values indicating the log_2_ fold change and *p*-value in the expression of migratory genes in LPS- and LPS + JQ1-treated cells are listed in Table S8. To validate the RNA-seq results, we confirmed the expression of the DEGs by qRT-PCR. JQ1 possesses anti-migratory activity, and we selected microglia migration-related genes (CSF2, IDO1, MMP3, MMP13, TNFSF10, and VCAM1) for qRT-PCR experiments (Fig. 4C). The mRNA levels of these migratory genes were upregulated in LPS-treated HMC3 cells but suppressed in LPS + JQ1-treated HMC3 cells. These data strongly suggest that JQ1 highly selectively suppresses gene expression during the inflammatory response and microglial migration.

### Differential expression of transcription factors in LPS- and JQ1-treated HMC3 cells

**Figure 5:**
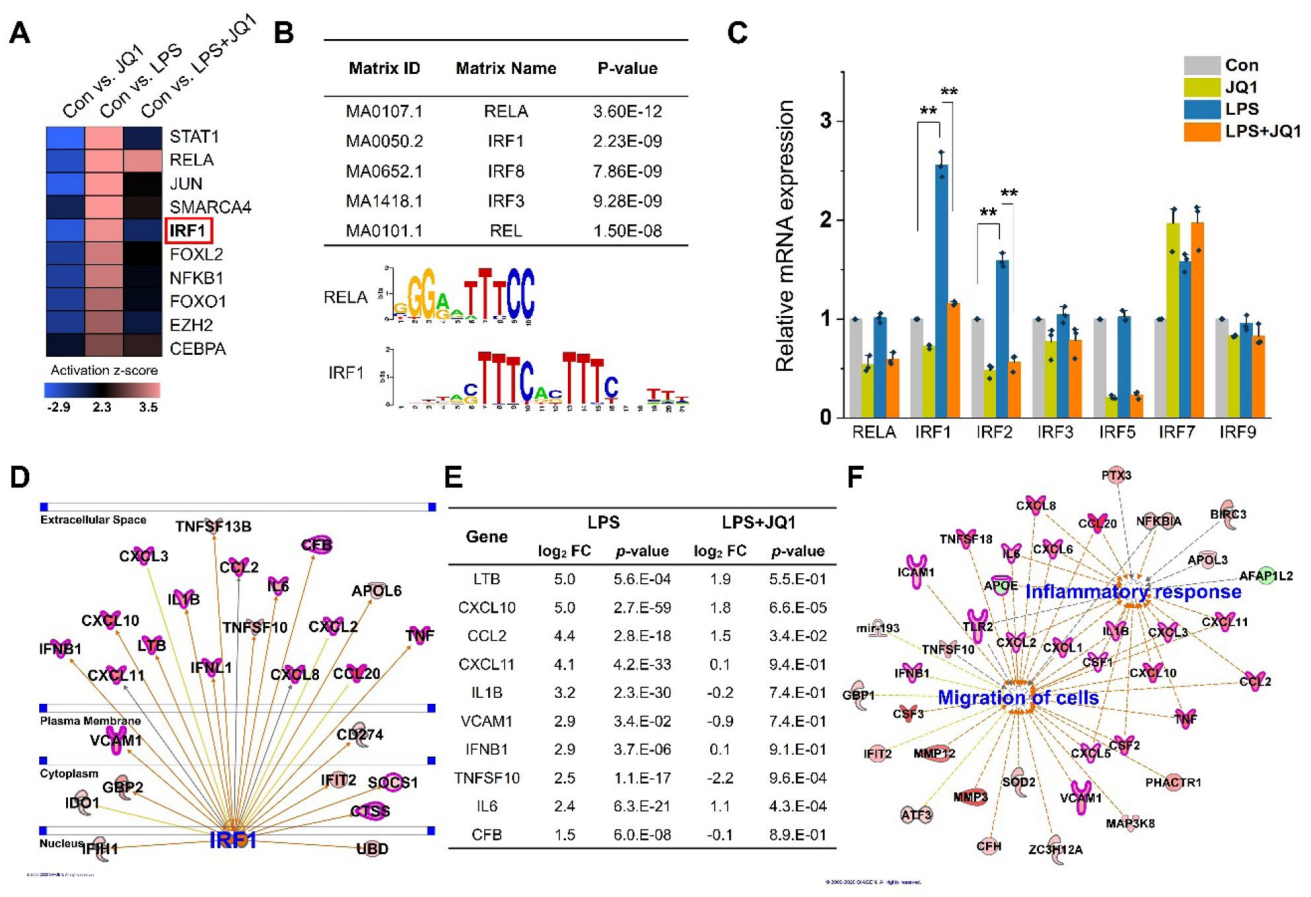
IRF1 regulates migration-related genes in LPS- and LPS + JQ1-treated HMC3 cells. (**A**) IPA upstream regulator analysis of DEGs in LPS-, JQ1- and LPS + JQ1-treated HMC3 cells. The color scale shown in the heat map represents the activation z-score value. (**B**) TF-binding motif analysis to determine TF-binding site enrichment in the promoters of migratory genes. Putative binding sites for IRF1was significantly enriched in LPS-induced migratory genes. (**C**) Expression of RELA and IRF family genes were analyzed by qRT-PCR and normalized to GAPDH transcript levels. Among these IRF family genes, IRF1 and IRF2 were significantly upregulated in LPS-treated cells. The data represent three independent experiments. The values are the mean ± SEM of triplicate experiments (**p < 0.001). (**D**) IPA upstream regulator analysis identified key regulatory gene IRF1, and it targets genes in LPS-treated cells. Of the genes regulated by IRF1, only migration-related genes are shown in bold and in red. The migration-related genes were highly correlated with IRF1. (**E**) Log_2_ fold changes and *p*-value in the expression of IRF1 target genes. (**F**) IPA network analysis of genes related to the inflammatory response and migration in LPS-treated cells. Of the genes identified, only chemotaxis-related genes are shown in bold and in red. Most inflammation-related genes overlap with migration-related genes.

To better understand the molecular signature, we focused on upstream regulators of DEGs in LPS + JQ1-treated HMC3 cells. Notably, the upregulated TFs in the LPS-treated cells, including STAT1, RELA, JUN, SMARCA4, and IRF1, showed a decreased activation z-score in LPS + JQ1-treated HMC3 cells (Fig. 5A). Also, we analyzed the TF-binding motif of DEGs related to the cell migration response. We perform the in silico analysis of *cis*-regulatory elements in promoter regions of coordinately regulated genes using PSCAN software. Assessment of the scores for the promoters of the DEGs (fold change ≥ 1.5 log_2_) revealed that the putative binding sites for RELA and IRF1 were significantly enriched (Fig. 5B). Among these TFs, we analyzed RELA and IRFs genes using qRT-PCR (Fig. 5C). We found that IRF1 and IRF2 were significantly upregulated in LPS-treated HMC3 cells but suppressed in LPS + JQ1-treated HMC3 cells. RELA and other IRFs, including IRF3, IRF5 IRF7, and IRF9, showed no significant LPS- and LPS + JQ1-treated HMC3 cells. Next, we used IPA to identify the target genes directly or indirectly regulated by IRF1 in LPS- and LPS + JQ1-treated HMC3 cells. Interestingly, we found that inflammatory genes, including cytokines, chemokines, and inflammation response genes (CCL2, CXCL10, IL1B, IFNL1, and CXCL11) and migratory genes (VCAM1) were directly regulated by IRF1 in LPS-treated cells (Fig. 5D). JQ1 suppressed the regulation of these inflammatory and migratory genes by IRF1 in LPS + JQ1-treated cells (Fig. 5E). The inflammation-related genes regulated by IRF1 overlap with the migration-related genes associated with the chemotaxis-related genes (Fig. 5F). Overall, these findings showed that the LPS-induced key TF IRF1 regulates inflammatory and migratory genes and that this regulation was suppressed by the decrease in IRF1 expression caused by JQ1.

### The transcription factor IRF1 regulated LPS-treated HMC3 cell migration

**Figure 6:**
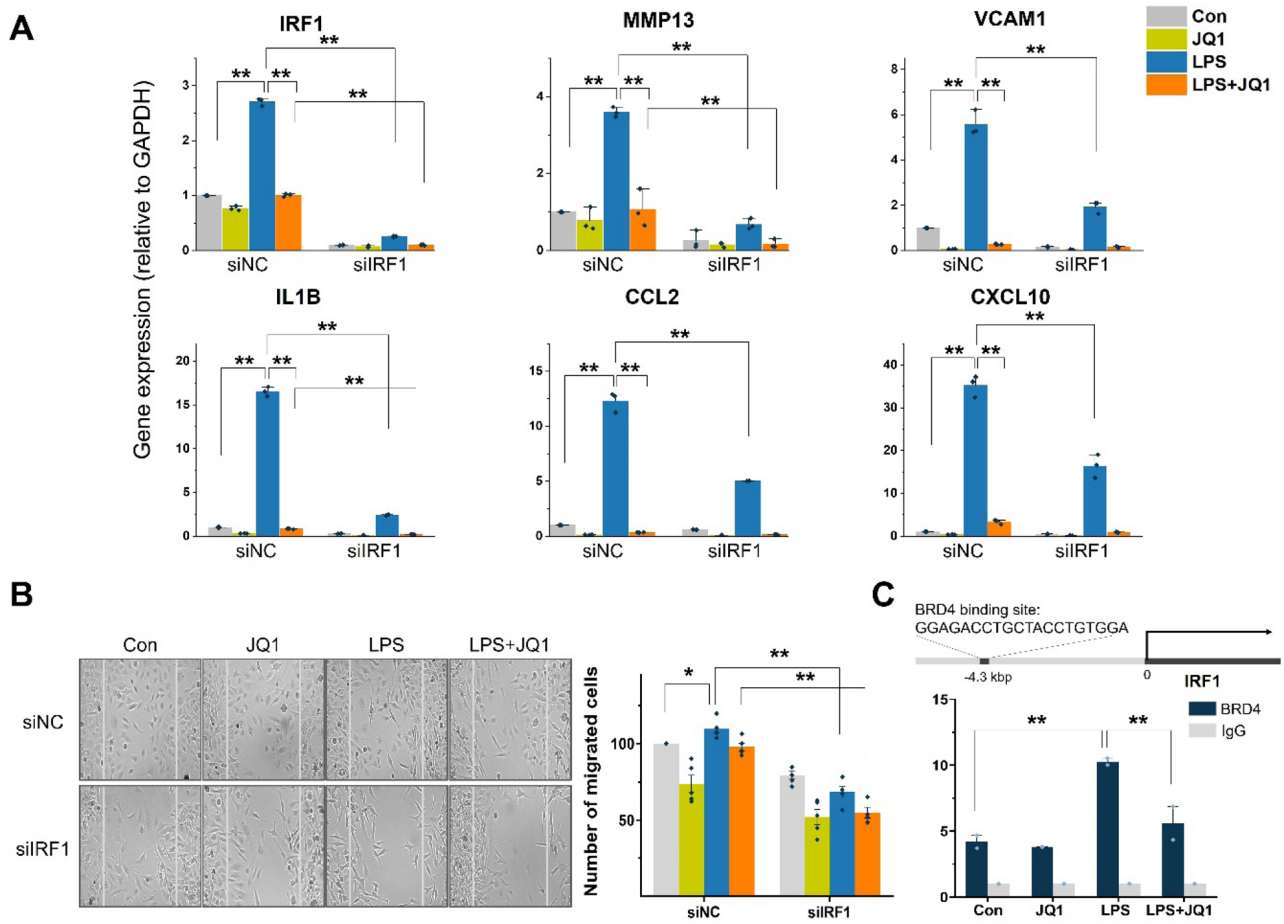
IRF1 regulates cell migration responses. (**A**) Expression of IRF1 target migration-related genes in siIRF1-treated cells were analyzed by qRT-PCR and normalized to GAPDH transcript levels. The knockdown of IRF1 significantly decreased migration-related gene expression levels. The data represent three independent experiments. The values are the mean ± SEM of triplicate experiments (**p < 0.001). (**B**) Representative images show the results of wound-healing/migration assay at 24 h after siIRF1 transfection in LPS- or JQ1-treated cells. The percentage of numbers of cells that migrated into the middle of the wound fields after 24 h of incubation are shown on the graph. Knockdown of IRF1 reduced the number of migrated cells. The data represent three independent experiments. The values are the mean ± SEM of triplicate experiments (*p < 0.05 and **p < 0.001). (**C**) ChIP-qPCR was conducted to examine whether BRD4 binds to IRF1 promoter. The ChIP-enriched samples were subjected to qPCR with specific primers targeting IRF1. Enrichment was calculated relative to control input DNA from three independent experiments. The values are the mean ± SEM of triplicate experiments (*p < 0.05 and **p < 0.001).

Furthermore, we hypothesized that IRF1 plays an essential role in the microglial cell migration response. To obtain direct experimental support of this hypothesis, we treated the cells with siRNA to selectively knockdown IRF1 expression and determine the effects on the migration response. Using scrambled siRNA (siNC; negative control group)-treated HMC3 cells, we found crucial cell migration genes, including MMP3 and VCAM1, and chemotaxis genes CCL2, CXCL10, and IL1B were increased after LPS treatment and decreased after LPS + JQ1 treatment. After LPS treatment, the expression of the IRF1 gene was increased in siNC-treated cells, but IRF1 siRNA (siIRF1)-treated HMC3 cells were remarkably reduced of IRF1 expression despite LPS treatment. Migration- and chemotaxis-related gene expression were shown the same results as IRF1. The knockdown of IRF1 led to a significant reduction in migration- and chemotaxis-related gene levels after LPS treatment (Fig. 6A). In addition, we performed an in vitro wound-healing assay to investigate the effects of IRF1 depletion on the migration of microglia. The LPS-treated cells slightly increased the number of cells that migrated to the wound field for 24 h. The LPS + JQ1-treated cells tended to decrease the number of migrated cells compared to LPS-treated cells, but this was not significant. siIRF1-treated cells significantly reduced the number of cells that migrated into the wound field compared with siNC-treated cells after LPS treatment (Fig. 6B). We deduced that BRD4/IRF1 binding might be involved in the regulation of inflammatory and migratory genes. Chromatin immunoprecipitation (ChIP)-qPCR was performed to verify this. The results revealed the BRD4 is directly bound to IRF1 promoter regions. Taken together, these results strongly suggest that LPS-induced IRF1 plays a role in the cell migration response in human microglia.

## Discussion

BET family proteins have emerged as crucial transcriptional regulators whose inhibition shows therapeutic activity against a wide range of different pathologies, particularly cancer and inflammation^13,15^. BRD4 can bind to acetylated lysine residues in both histones and transcription factors using its bromodomains. Previous studies reported that BRD4 plays an essential role in modulating inflammatory response after spinal cord injury in rats. BRD4 was expressed in microglia, and the expression of BRD4 was increased in inflammation-induced cells. Pro‐inflammatory cytokines promote the level of BRD4 in microglia^18^. In addition, the relationship between BRD4 and IRF1 was reported in a previous study, and it was verified that IRF1 directly interacts with BRD4 using co-immunoprecipitation and ChIP assays^22^. BRD4 forms a transcriptional complex with IRF1, RNA polymeraseII, and P-TEFb and regulates the MLKL gene expression. JQ1 treatment interferes with the formation of the transcriptional complexes of the MLKL gene by disrupting the association between BRD4 and IRF1. Also, Hogg et al. reported that BRD4 and IRF1 co-regulate interferon-induced immune checkpoint ligand PD-L1 transcription^23^. Based on these results, we suggest that IRF1 and BRD4 regulate LPS-induced inflammatory and migratory genes, and JQ1 effectively inhibits them.

BET inhibitors have exhibited broad anti-inflammatory and anti-cancer activities and have been tested in human clinical trials. For example, synthetic BET inhibitor (I-BET) suppressed the expression of essential inflammatory genes and was shown to have the ability to treat inflammation in vivo^24^. JQ1 disrupts the interaction of BET proteins with acetylated histones and results in the loss of enhancer-promoter communications. According to the transcriptomic analysis of BV-2 microglial cells, JQ1 could be inhibited the expression of several LPS-inducible proinflammatory genes^19^. In animal experiments, JQ1 attenuated LPS-induced microglial activation and neuroinflammation in vivo^25^. In the present study, we demonstrated that LPS-induced inflammatory and migratory transcripts in human microglia were effectively inhibited by the BET inhibitor JQ1. However, JQ1 appears to be marginal or ineffective on the LPS-induced inflammatory gene. In the case of the C-X-C motif chemokine ligand, other CXCL families were increased by JQ1 except for CXCL8, CXCL10, CXCL11, and TNF and NFKBIA were also increased. These results are consistent with our previous transcriptome analysis studies using the mouse bone marrow-derived macrophage^19,20^. Also, Nicodeme et al. reported that some cytokines and chemokines such as Tnf, Ccl2-5, and Cxcl1/2 were unaffected I-BET in bone marrow-derived macrophages, indicating a highly selective effect of I-BET on LPS-inducible gene expression. The selectivity of gene responses to I-BET correlated with epigenetic states and LPS-induced gene activation^24^. Further studies are required to clarify the selectivity and anti-inflammatory potential of JQ1.

Recent studies have indicated that microglial cell lines differ both genetically and functionally from primary microglia and ex vivo microglia^26,27^. Despite these limitations, microglia cell lines are suitable for biochemical and molecular approaches and high-throughput screening assays, requiring high cell numbers. Furthermore, microglia studies using human cells have been limited due to the limitation of primary sources of human microglia and the difficulty of obtaining sufficient human microglia numbers^28^. Nagai et al. reported that the Immortalized human microglial cell line HMO6 had similar properties to human microglia^29^. Inconsistent with our results, HMC3 cells showed a similar expression pattern of cytokines and chemokines such as CCL2, IL-1β, IL-6, IL-10, and TNFa compared with human primary microglia and HMO6 cells by the same concentration of 100 ng/ml LPS treatment. HMC3 cells are verified human microglia cell lines^30^, we also validated microglial cells using microglia-lineage markers.

Chemokines play an essential role in the attraction and activation of immune cells and the modulation of synaptic activity and survival of neurons^31^. The CC chemokine ligand (CCL) is secreted by inflammatory signals to produce a gradient that attracts monocytes to the site of inflammation. CCL2 plays a role in neuroinflammatory diseases through glial neuroinflammatory changes^32^. The crucial role of C-X-C motif chemokine ligand 10 (CXCL10) occurs through its association with chronic Th1-type inflammatory diseases. CXCL10 regulates immune responses through the activation and recruitment of leukocytes such as T cells and monocytes/macrophages^33^. The secretion of matrix metallopeptidases (MMPs) is one of the hallmarks of microglial activation and migration. LPS-activated microglia and monocytes contain increased proinflammatory cytokine levels, inducing the secretion of diverse MMPs^34^. VCAM1 is a member of the immunoglobulin superfamily. Its expression is increased during the inflammatory response due to inflammatory cytokines such as tumor necrosis factor (TNF)^35,36^.

The active migration of microglial cells is closely associated with inflammatory responses^3,12^. As a result of inflammatory stimuli, microglia and resident innate immune cells produce inflammatory cytokines. Cytokines promote the increased motility of microglia. Activated microglia directly migrate to the site of injury using a chemoattractant gradient. These cytokines induce further production of chemokines and can alter the chemokine gradient, ultimately changing the chemokine environment^37^. Chemokines have been shown to induce chemotaxis in microglia/monocytes. These induce migration and a proinflammatory phenotype in microglia^38,39^. Intracellular signals regulate microglial processes' motility through various signaling pathways involved in microglial chemotaxis^11,40^. Therefore, increased migration may also be a feature of activated microglial cells.

Based on TFs analysis, we found that LPS significantly upregulated IRF1 in human microglia (Fig. 5C). The results in mouse microglia, in which most IRF gene families were upregulated by LPS^41,42^. Therefore, we hypothesized that IRF1 regulates migration- and chemotaxis-related genes. The knockdown of IRF1 using siRNA significantly downregulated the expression of migration-related genes (MMP13 and VCAM1) and chemotaxis-related genes (IL1B, CCL2, and CXCL10) and inhibited microglial migration.

IFN regulatory factors (IRFs) play a vital role in antiviral defense, innate immunity, cell differentiation, and apoptosis^43^. Several IRFs, including IRF1, are involved in type I IFN gene transcription. Previous studies reported that IRF1 contributes to IFN-regulated genes (IRGs) in immune system regulation^44,45^. In chronic inflammatory autoimmune disease mouse models, IRF1 expression was associated with the expression of IFNβ, leading to activation of the JAK-STAT pathway. The JAK-STAT pathway's blockade reduced the expression of IRGs^46^. Further studies are required to clarify the detailed mechanisms by which IRF1 regulates microglial migration.

Overall, the results of genome-wide RNA-seq analysis revealed that JQ1 significantly inhibited LPS-induced inflammatory and migratory genes. However, since JQ1 has a broad target spectrum for BET proteins^47^, further studies will be required to investigate the mechanism by which bromodomains are involved in inflammation in microglia using the first (BD1) and second (BD2) bromodomains-specific inhibitors. First-generation pan-BET inhibitors are effectively inhibited by BRD and can induce clinical remission. However, toxicity, side effects, and the short duration of their clinical response have limited their broad therapeutic application^13^. Recently developed BD1- and BD2-selective inhibitors such as GSK078, GSK046, and ABBV-744 were shown to be highly potent and selective inhibitors of the bromodomains. The BD1 inhibitor GSK078 showed comparable efficacy to the pan-BET inhibitor in cancer models. The BD2 inhibitors GSK046 and ABBV-744 were predominantly effective in inflammatory and autoimmune disease models^48,49^. We are understudying BD2-specific inhibitors to understand the mechanisms by which bromodomains act on inflammation and migration in microglia.

## Conclusions

In this study, we performed transcriptome analyses of inflammation and migration in human microglial HMC3 cell line. We found that the BET inhibitor JQ1 played a critical role in modulating inflammatory and migratory responses after LPS-induced inflammation. We confirmed that JQ1 attenuates proinflammatory chemokine, cytokine, and interferon response genes in LPS-treated HMC3 cells and that LPS promotes microglial migration, whereas JQ1 inhibits migration by regulating the expression of the IRF1. These findings suggest that JQ1 modulates the functional activities of immune cells, thus exert immunosuppressive effects by inhibiting chemokine and cytokine production, and selectively inhibits the expression of inflammation- and migration-related genes to exert its anti-inflammatory and anti-migratory functions.

## Materials and methods

### Cell culture and treatment

HMC3 human microglial cells were purchased from the Korean Cell Line Bank (Seoul, Korea). The cells were cultured in minimum essential medium (MEM) supplemented with 100 IU/ml penicillin, 10 μg/ml streptomycin, and 10% fetal bovine serum (FBS) (Thermo Fisher Scientific, Waltham, MA, USA) and were maintained in a humidified incubator at 37 °C with 95% air/5% CO_2_. The cells were treated with 10 ng/ml to 1 μg/ml LPS (Sigma-Aldrich, St. Louis, MO, USA) for 4 h to 24 h under standard culture conditions. A stock solution (10 mM) of JQ1 (Tocris Bioscience, Minneapolis, MN, USA) was dissolved in dimethyl sulfoxide (DMSO; Sigma-Aldrich, St. Louis, MO, USA) and then diluted in DMEM for experiments. The cells were treated with 50 nM to 5 μM JQ1 with LPS (100 ng/mL) for 4 h to 24 h under standard culture conditions.

### Total RNA extraction and quantitative RT-PCR

Total RNA extractions and cDNA sample preparation were using Takara's kits according to the manufacture's instruction (Takara, Shiga, Japan). Quantitative Reverse Transcription PCR (qRT-PCR) was performed using an ABI 7500 real-time PCR system (Applied Biosystems Inc., Foster City, CA, USA). The critical threshold ($\Delta$CT) value was normalized to the expression of glyceraldehyde-3-phosphate dehydrogenase (GAPDH), which served as an internal control. The results were also analyzed using the comparative critical threshold ($\Delta \Delta$CT) method^19^. Specific primers were designed using Primer Bank (http://pga.mgh.harvard.edu/primerbank/index.html). The primers for qRT-PCR are listed in Table 1.

**Table 1:** List of primers for qRT-PCR.

Gene	Forward (5′ → 3′)	Reverse (5′ → 3′)
CCL2	CAG CCA GAT GCA ATC AAT GCC	TGG AAT CCT GAA CCC ACT TCT
CSF2	CCG GAA ACT TCC TGT GCA AC	GTC TCA CTC CTG GAC TGG CT
CXCL8	CAC TGC GCC AAC ACA GAA AT	TTC TCA GCC CTC TTC AAA AAC TTC
CXCL10	GTG GCA TTC AAG GAG TAC CTC	TGA TGG CCT TCG ATT CTG GAT T
IDO1	GCC AGC TTC GAG AAA GAG TTG	ATC CCA GAA CTA GAC GTG CAA
IFIT2	AAG CAC CTC AAA GGG CAA AAC	TCG GCC CAT GTG ATA GTA GAC
IFNB1	TCT CCT GTT GTG CTT CTC CAC	GCC TCC CAT TCA ATT GCC AC
IL1B	ATG ATG GCT TAT TAC AGT GGC AA	GTC GGA GAT TCG TAG CTG GA
IL6	TCC TTC TCC ACA AAC ATG TAA CAA	TCA CCA GGC AAG TCT CCT CA
IRF1	ATG CCC ATC ACT CGG ATG C	CCC TGC TTT GTA TCG GCC TG
IRF2	AAT GCT GCC CCT ATC AGA ACG	CAG GAC CGC ATA CTC AGG AGA
IRF3	AGA GGC TCG TGA TGG TCA AG	AGG TCC ACA GTA TTC TCC AGG
IRF5	GGG CTT CAA TGG GTC AAC	GCC TTC GGT GTA TTT CCC TG
IRF7	CCC ACG CTA TAC CAT CTA CCT	GAT GTC GTC ATA GAG GCT GTT G
IRF9	GCC CTA CAA GGT GTA TCA GTT G	TGC TGT CGC TTT GAT GGT ACT
MMP3	CGG TTC CGC CTG TCT CAA G	CGC CAA AAG TGC CTG TCT T
MMP13	TCC TGA TGT GGG TGA ATA CAA TG	GCC ATC GTG AAG TCT GGT AAA AT
RELA	CCA TTG AGC GGA AGA TTC AAC T	CTG CTG GTC CCG ATA TGA GG
TNFSF10	TGC GTG CTG ATC GTG ATC TTC	GCT CGT TGG TAA AGT ACA CGT A
VCAM1	ATG CCT GGG AAG ATG GTC G	GAC GGA GTC ACC AAT CTG AGC

### Cell viability assay

A cell viability assay was performed using a PreMix water soluble tetrazolium-1 (WST-1) cell proliferation assay kit (Takara, Shiga, Japan). Cells were seeded at a density of 1 × 10^4^ per well in 96-well plates and treated with LPS or JQ1 for 0 to 48 h. After incubation, PreMix WST-1 was added to each well, and incubated at 37 °C for 4 h, after which the absorbance of cells at 450 nm was measured using microplate reader. The data represent three independent experiments (n = 3).

### RNA library preparation and RNA-seq

Total RNA was extracted from samples taken from 4 groups (12 independent samples) of HMC3 cells: DMSO-treated HMC3 cells (Con, 3 samples), LPS-treated HMC3 cells (LPS, 3 samples), JQ1-treated HMC3 cells (JQ1, 3 samples), and LPS + JQ1-treated HMC3 cells (LPS + JQ1, 3 samples). The RNA library used for RNA-seq was created as previously described^19^. Briefly, total RNA was extracted using RNAiso Plus (Takara, Shiga, Japan) and the RNeasy Mini Kit (QIAGEN Inc., Hilden, Germany). Ribosomal RNA (rRNA) was depleted by the RiboMinus Eukaryote kit (Invitrogen, Carlsbad, CA, USA). RNA libraries were created using the NEBNext Ultra Directional RNA Library Preparation Kit for Illumina (New England BioLabs, Ipswich, MA, USA). Transcriptome sequencing was performed using the Illumina HiSeq2500 platform (Macrogen, Seoul, Korea).

### Differential gene expression analysis

To analyze the differentially expressed genes (DEGs), we adopted a previously reported pipeline^50^. Briefly, FASTQ data from RNA-seq experiments were checked the quality and trimmed with the Trimmomatic^51^. The sequence read archive FASTQ files were aligned to the *Homo sapiens* reference sequence GRCh38 using STAR (version 2.7.1) alignment software^52^. DESeq2^53^ was used with the default parameters to obtain DEGs. DESeq2 was used to normalize for sequencing depth and RNA composition using the median of ratios method. False discovery rate (FDR) < 0.01, *p* ≤ 0.05, and fold change in expression ≥ 1.5 log_2_ were used as the criteria to define DEGs. The RNA-seq data were deposited in the Gene Expression Omnibus database under dataset accession No. GSE155408.

### Functional annotation and canonical pathway analysis of the datasets

Database for Annotation, Visualization, and Integrated Discovery (DAVID) (version 6.8) software (http://david.abcc.ncifcrf.gov/home.jsp) was used to determine functions most significantly enriched in the genes in the datasets^54^. DAVID uses a modified Fisher's exact *p-*value to examine gene ontology (GO) enrichment. FDR < 0.05 was used as the criteria for GO term analysis. Values less than 0.05 are considered to indicate enrichment in the annotation category. To analyze the genetic networks and pathways involved in the effects of JQ1, genes in the RNA-seq datasets with a cutoff *p* ≤ 0.05, and fold change in expression ≥ 1.5 log_2_ from the LPS- and JQ1-treated HMC3 cells were used. Each of these genes was mapped to objects by Ingenuity Pathway Analysis^55^ (IPA; QIAGEN Inc., Hilden, Germany, https://www.qiagenbioinformatics.com/products/ingenuitypathway-analysis). IPA software conducts functional analysis to show genes involved in biological functions and disease.

### Upstream regulator analysis

The expression and predicted target genes of TFs in response to LPS or LPS + JQ1 treatment were analyzed using upstream regulator analysis with IPA software. The genes from the datasets that were shown to be associated with canonical pathways by IPA were assessed by predicted target gene analysis of the identified TFs. The *p*-values were calculated with Fisher's exact test reflected the probability that an association could be explained by chance.

### Analysis of transcription factor binding motif enrichment

GenBank accession numbers were used to analyze TF binding motifs that conducted by PSCAN software^56^. The JASPAR 2016 database, which analyzes of TF binding profile of the promoter regions, ranged from 950 base pair (bp) downstream of the transcription start site to + 50 bp^57^.

### Knockdown of IRF1 gene expression by siRNA treatment

Small interfering RNA (siRNA) specific for IRF1 was purchased from Ambion by Thermo Fisher (Waltham, MA, USA). IRF1 was attenuated with the following siRNA strands: sense strand, 5′-GCAGAUUAAUUCCAACCAAtt-3′, and antisense strand, 5′-UUGGUUGGAAUUAAUCUGCat-3′. HMC3 cells were transfected with the IRF1 siRNA constructs or nontargeting siRNAs (Ambion by Thermo Fisher, Waltham, MA, USA) using Lipofectamine RNAiMAX transfection reagent (Invitrogen, Carlsbad, CA, USA) following the Silencer Select siRNA transfection protocol. IRF1 siRNA was used at concentrations of 25 nM, and transfection was carried out for 48 h.

### Chromatin immunoprecipitation (ChIP)-qPCR

ChIP assay was performed as previously described^58^. The 2 × 10^7^ cells were collected and immunoprecipitated with antibodies against IRF1 (Santa Cruz Biotechnology, Inc., Dallas, TX, USA; sc- 514,544) and normal mouse IgG (Santa Cruz Biotechnology, Inc., Dallas, TX, USA) used as a control with Dynabeads Protein A beads (Invitrogen, Carlsbad, CA, USA). The immunoprecipitated DNA was extracted and analyzed by real-time quantitative PCR. The ChIP-qPCR data were normalized to the amounts of input DNA. The data represent three independent experiments. Primers used for ChIP-PCR were described as follows; forward: 5′-GGAGACCTGCTACCTGTGGA-3′ and reverse: 5′-ACTTCTGGACCTGGAACCTG-3′.

### Wound-healing/migration assay

The wound-healing assay was performed as previously described^58^. Briefly, the cells were seeded into the ibidi culture-inserts wells (ibidi, Martinsried, Germany) and incubated for 24 h. After incubation, the cells were treated with either LPS or JQ1 for 4 h. Then, the inserts were removed, and the cells were incubated for 24 h. The cells were visualized using a microscope (Leica, Wetzlar, Germany). After 0, 24, and 48 h of incubation, cells that migrated to the wound field were counted.

### Statistical analyses

All data are expressed as the mean ± standard error of the mean (SEM). The statistical analyses were performed using IBM SPSS Statistics ver. 26.0 (IBM Corporation, Armonk, NY, USA). All qRT-PCR and ChIP-qPCR data were tested using one-way ANOVA followed by Tukey's honestly significant difference (HSD) post hoc test. The wound-healing migration assay data were tested using one-way ANOVA followed by Tukey's HSD post hoc test. Differences for which *p* < 0.05 were considered significant.

## Supplementary Information

Supplementary Information 1.

Supplementary Information 2.
