# Supplementary material for: The BET inhibitor attenuates the inflammatory response and cell migration in human microglial HMC3 cell line
Source: Sci Rep. 2021 Apr 23;11:8828. doi: 10.1038/s41598-021-87828-1 (PMC8065145; doi:10.1038/s41598-021-87828-1)
Supplement: Supplementary file 1 — Supplementary Information 1. [file 41598_2021_87828_MOESM1_ESM.docx]

**Supplementary Figure Legends**

**
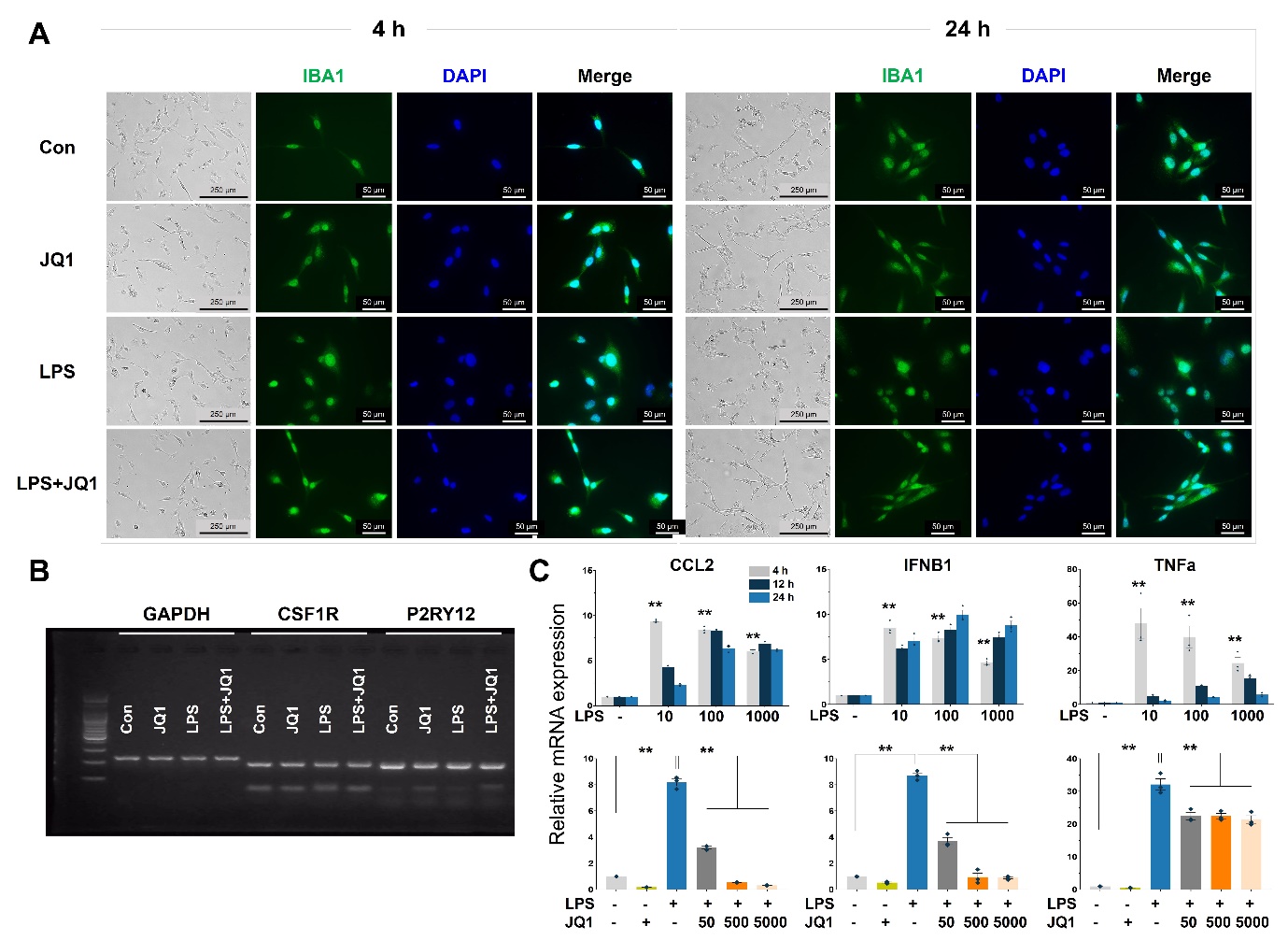
**

**Figure S1. Identification of human microglial cell line HMC3.**

(A) HMC3 cells were cultured for 4 h and 24 h after treatment with LPS (100 ng/ml) and JQ1 (500 nM) and stained for IBA1 (green fluorescence). Nuclei were counterstained with DAPI (blue). (B) The full-length agarose gel electrophoresis image of mRNA expression of other microglia-lineage markers (P2RY12 and CSF1R). GADPH primer was used for as internal control. (C) HMC3 cells were treated with LPS at different doses (10, 100, and 1000 ng/ml) for 4 h. Inflammatory genes were significantly upregulated in cells treated with LPS compared to DMSO-treated control cells (upper panel). Inflammatory genes were significantly downregulated in cells treated with JQ1 (bottom panel). Gene expression was normalized to GAPDH transcript levels. The data represent three independent experiments. The values are the mean ± SEM of triplicate experiments (* *p* < 0.05 and ** *p* < 0.001).

**
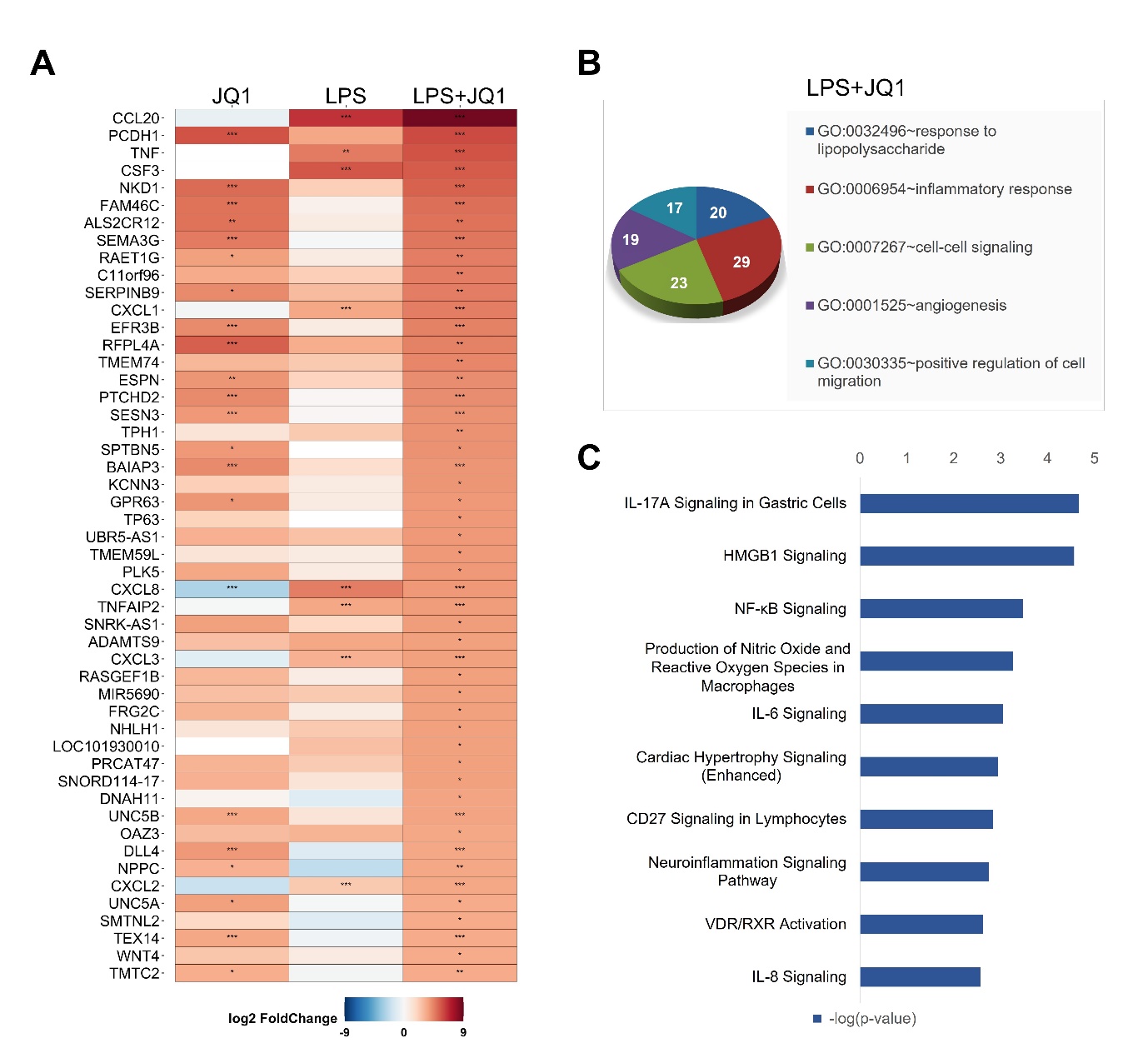
**

**Figure S2. Differentially expressed genes in LPS+JQ1-treated HMC3 cells.**

(A) Heat map showing expression of the top 50 upregulated genes in LPS+JQ1-treated HMC3 cells at 4 h determined by RNA-seq (*p* ≤ 0.05 and fold change ≥ 1.5 log_2_). Each experiment was performed in experimental triplicates (n = 3) for each condition, and the results were individually combined. The color scale shown in the heat map represents the log2 fold change values. (B) GO term analysis of the biological processes associated with up- and downregulated genes after LPS+JQ1 treatment for 4 h. GO analysis of the number of genes shown in the van diagram. (C) Biological pathway analysis of DEGs in cells treated with LPS+JQ1 using IPA showed up- and downregulated genes.

**
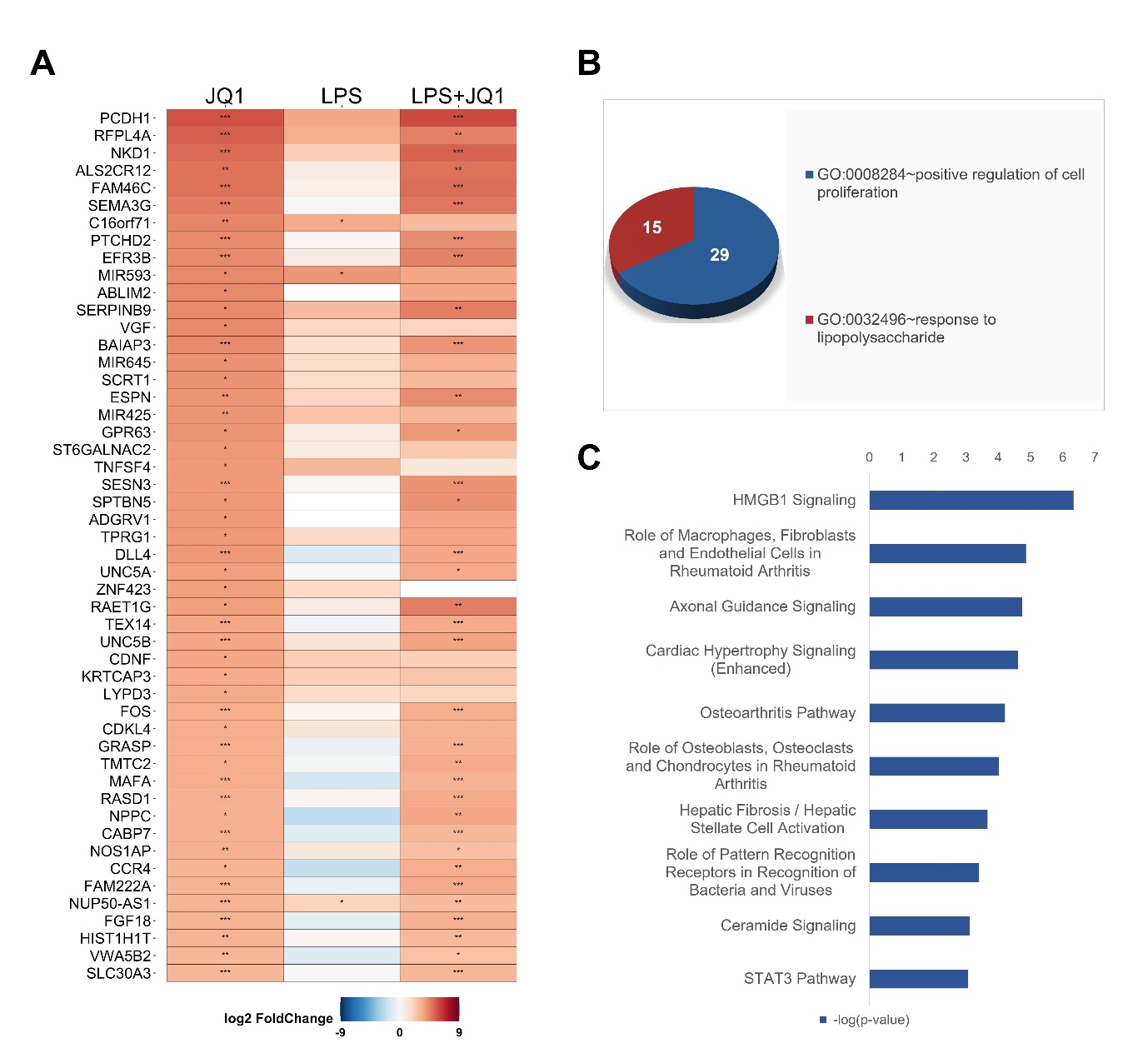
**

**Figure S3. Differentially expressed genes in JQ1-treated HMC3 cells.**

(A) Heat map showing expression of the top 50 upregulated genes in JQ1-treated HMC3 cells at 4 h determined by RNA-seq (*p* ≤ 0.05 and fold change ≥ 1.5 log_2_). Each experiment was performed in experimental triplicates (n = 3) for each condition, and the results were individually combined. The color scale shown in the heat map represents the log2 fold change values. (B) GO term analysis of the biological processes associated with up- and downregulated genes after JQ1 treatment for 4 h. GO analysis of the number of genes shown in the van diagram. (C) Biological pathway analysis of DEGs in cells treated with JQ1 using IPA showed up- and downregulated genes.
