# Supplementary material for: The BET inhibitor attenuates the inflammatory response and cell migration in human microglial HMC3 cell line
Source: Sci Rep. 2021 Apr 23;11:8828. doi: 10.1038/s41598-021-87828-1 (PMC8065145; doi:10.1038/s41598-021-87828-1)
Supplement: Supplementary file 2 — Supplementary Information 2. [file 41598_2021_87828_MOESM2_ESM.docx]

**Title: The BET inhibitor attenuates the inflammatory response and cell migration in human microglial cell line HMC3**

Mina Baek^1,2‡^, Eunyoung Yoo^3‡^, Hae In Choi^3^, Ga Yeong An^3^, Jin Choul Chai^4^, Young Seek Lee^4**^, Kyoung Hwa Jung^5**^, Young Gyu Chai^1,3*^

^1^ Department of Molecular & Life Science, Hanyang University, Ansan, 15588, Republic of Korea

^2^ Institute of Natural Science and Technology, Hanyang University, Ansan, 15588, Republic of Korea

^3^ Department of Bionanotechnology, Hanyang University, Seoul, 04673, Republic of Korea

^4^ College of Veterinary Medicine, Seoul National University, Seoul, 08826, Republic of Korea

^5^ Department of biopharmaceutical system, Gwangmyeong Convergence Technology Campus of Korea Polytechnic II, Incheon, 21417, Republic of Korea

^‡^ Contributed equally.

^*^ Corresponding Author.

^**^Co-Corresponding Author.

**Table S1. Top 50 significant upregulated genes in LPS-treated HMC3 cells.**

| Gene Accession_ID | Gene Symbol | log_2_FoldChange | *p*-value |
| --- | --- | --- | --- |
| NM_004591 | CCL20 | 6.5 | 1.6.E-07 |
| NM_001178147 | CSF3 | 5.6 | 5.1.E-05 |
| NM_002422 | MMP3 | 5.5 | 7.2.E-05 |
| NM_009588 | LTB | 5.0 | 5.6.E-04 |
| NM_001565 | CXCL10 | 5.0 | 2.7.E-59 |
| NM_000594 | TNF | 4.7 | 1.8.E-03 |
| NM_000584 | CXCL8 | 4.6 | 0.0.E+00 |
| NM_005092 | TNFSF18 | 4.6 | 3.4.E-03 |
| NM_001781 | CD69 | 4.5 | 3.7.E-03 |
| NC_000005.10 | MIR146A | 4.4 | 6.0.E-03 |
| NM_002982 | CCL2 | 4.4 | 2.8.E-18 |
| NM_002426 | MMP12 | 4.3 | 9.1.E-03 |
| NM_001077182 | FSCN2 | 4.3 | 1.1.E-02 |
| NC_000003.12 | ZNF197-AS1 | 4.2 | 1.3.E-02 |
| NM_001302123 | CXCL11 | 4.1 | 4.2.E-33 |
| NC_000007.14 | MIR593 | 4.0 | 1.7.E-02 |
| NM_001160419 | ZBP1 | 4.0 | 6.8.E-03 |
| NM_000758 | CSF2 | 4.0 | 1.5.E-08 |
| NM_001013624 | ZNF385C | 3.9 | 2.8.E-02 |
| NM_001100 | ACTA1 | 3.8 | 3.6.E-02 |
| NM_001243531 | UBE2Q2L | 3.8 | 3.6.E-02 |
| NC_000004.12 | CETN4P | 3.7 | 4.3.E-02 |
| NM_002993 | CXCL6 | 3.6 | 1.0.E-03 |
| NM_001511 | CXCL1 | 3.5 | 1.1.E-46 |
| NM_001142316 | LMO2 | 3.5 | 1.1.E-05 |
| NM_006291 | TNFAIP2 | 3.5 | 8.1.E-73 |
| NM_001322296 | SIDT1 | 3.4 | 6.7.E-03 |
| NM_052942 | GBP5 | 3.4 | 2.6.E-12 |
| NM_139170 | C16orf71 | 3.4 | 3.2.E-02 |
| NC_000001.11 | GBP1P1 | 3.3 | 2.3.E-03 |
| NM_000576 | IL1B | 3.2 | 2.3.E-30 |
| NM_001561 | TNFRSF9 | 3.2 | 1.3.E-21 |
| NM_002090 | CXCL3 | 3.2 | 5.7.E-06 |
| NM_172140 | IFNL1 | 3.2 | 1.7.E-02 |
| NM_004120 | GBP2 | 3.1 | 3.9.E-05 |
| NM_004054 | C3AR1 | 3.1 | 2.4.E-06 |
| NM_153372 | C1QTNF1 | 3.0 | 3.6.E-07 |
| NM_001322314 | PHACTR1 | 3.0 | 2.5.E-02 |
| NM_001285485 | NEURL3 | 2.9 | 3.7.E-04 |
| NM_001078 | VCAM1 | 2.9 | 3.4.E-02 |
| NM_002176 | IFNB1 | 2.9 | 3.7.E-06 |
| NM_001190945 | TRAF1 | 2.8 | 7.1.E-50 |
| NM_001040429 | PCDH17 | 2.8 | 1.5.E-03 |
| NM_052941 | GBP4 | 2.7 | 2.0.E-17 |
| NM_004428 | EFNA1 | 2.7 | 8.0.E-22 |
| NM_002852 | PTX3 | 2.7 | 4.3.E-225 |
| NM_182962 | BIRC3 | 2.6 | 2.4.E-23 |
| NM_002427 | MMP13 | 2.6 | 1.7.E-03 |
| NM_002575 | SERPINB2 | 2.6 | 9.3.E-05 |
| NM_001114735 | BCL2A1 | 2.5 | 6.2.E-09 |

**Table S2. Top 13 significant downregulated genes in LPS-treated HMC3 cells.**

| **Gene Accession_ID** | **Gene Symbol** | **log_2_FoldChange** | ***p*-value** |
| --- | --- | --- | --- |
| NM_001270584 | IQCA1 | -4.302 | 1.1.E-02 |
| NM_002457 | MUC2 | -3.997 | 2.3.E-02 |
| NR_029376 | LOC100294362 | -3.900 | 1.0.E-02 |
| NM_001286086 | C11orf98 | -3.769 | 3.9.E-02 |
| NM_003813 | ADAM21 | -2.781 | 4.7.E-02 |
| NM_001284308 | ADAP1 | -2.723 | 5.3.E-02 |
| NM_207382 | UBE2Q2P1 | -2.378 | 5.5.E-02 |
| NM_001003927 | EVI2A | -2.173 | 1.7.E-03 |
| NR_003194 | SNORD114-2 | -2.079 | 2.3.E-02 |
| NM_000839 | GRM2 | -2.077 | 2.2.E-02 |
| NR_030345 | MIR614 | -1.933 | 1.9.E-02 |
| NM_001102601 | CCDC163P | -1.624 | 5.4.E-02 |
| NR_002745 | SNORD48 | -1.606 | 3.2.E-02 |
| NR_039746 | MIR4521 | -1.537 | 1.5.E-02 |
| NM_020225 | STOX2 | -1.533 | 4.9.E-02 |
| NM_000041 | APOE | -1.510 | 5.4.E-02 |
| NM_014310 | RASD2 | -1.502 | 7.7.E-03 |
| NM_001001936 | AFAP1L2 | -1.473 | 3.1.E-02 |

**Table S3. Top 50 significant upregulated genes in LPS+JQ1-treated HMC3 cells.**

| Gene Accession_ID | Gene Symbol | log_2_FoldChange | *p*-value |
| --- | --- | --- | --- |
| NM_004591 | CCL20 | 8.7 | 2.7.E-13 |
| NM_001278615 | PCDH1 | 5.9 | 1.6.E-05 |
| NM_006291 | TNF | 5.7 | 4.7.E-05 |
| NM_001178147 | CSF3 | 5.5 | 7.2.E-05 |
| NM_033119 | NKD1 | 5.3 | 2.0.E-04 |
| NM_017709 | FAM46C | 5.0 | 1.1.E-30 |
| NM_001289993 | ALS2CR12 | 4.9 | 1.1.E-03 |
| NM_020163 | SEMA3G | 4.8 | 4.5.E-04 |
| NM_001001788 | RAET1G | 4.6 | 3.2.E-03 |
| NM_001145033 | C11orf96 | 4.6 | 3.4.E-03 |
| NM_004155 | SERPINB9 | 4.6 | 4.9.E-03 |
| NM_001511 | CXCL1 | 4.6 | 1.1.E-89 |
| NM_014971 | EFR3B | 4.5 | 2.3.E-22 |
| NM_001145014 | RFPL4A | 4.5 | 6.7.E-03 |
| NM_153015 | TMEM74 | 4.4 | 1.8.E-03 |
| NM_031475 | ESPN | 4.2 | 3.6.E-03 |
| NC_000001.11 | PTCHD2 | 4.2 | 6.1.E-04 |
| NM_144665 | SESN3 | 4.1 | 5.3.E-17 |
| NM_004179 | TPH1 | 4.1 | 6.2.E-03 |
| NM_016642 | SPTBN5 | 4.1 | 2.0.E-02 |
| NM_001199097 | BAIAP3 | 4.0 | 2.8.E-08 |
| NM_001204087 | KCNN3 | 4.0 | 2.7.E-02 |
| NM_001329148 | TP63 | 3.9 | 2.8.E-02 |
| NM_001143957 | GPR63 | 3.9 | 2.8.E-02 |
| NC_000008.11 | UBR5-AS1 | 3.9 | 3.0.E-02 |
| NM_012109 | TMEM59L | 3.9 | 2.9.E-02 |
| NM_001243079 | PLK5 | 3.9 | 3.2.E-02 |
| NM_000584 | CXCL8 | 3.9 | 1.1.E-226 |
| NM_006291 | TNFAIP2 | 3.8 | 5.5.E-93 |
| NC_000003.12 | SNRK-AS1 | 3.8 | 4.2.E-02 |
| NM_001318781 | ADAMTS9 | 3.7 | 4.4.E-02 |
| NM_002090 | CXCL3 | 3.7 | 1.7.E-08 |
| NM_152545 | RASGEF1B | 3.7 | 1.5.E-02 |
| NC_000006.12 | MIR5690 | 3.7 | 4.2.E-02 |
| NM_005598 | NHLH1 | 3.7 | 4.5.E-02 |
| NM_001124759 | FRG2C | 3.7 | 4.5.E-02 |
| NC_000006.12 | LOC101930010 | 3.7 | 4.7.E-02 |
| NC_000016.10 | PRCAT47 | 3.7 | 4.4.E-02 |
| NC_000014.9 | SNORD114-17 | 3.7 | 1.8.E-02 |
| NM_001277115 | DNAH11 | 3.6 | 2.7.E-02 |
| NM_001244889 | UNC5B | 3.6 | 6.5.E-07 |
| NM_001301371 | OAZ3 | 3.5 | 2.5.E-02 |
| NM_019074 | DLL4 | 3.5 | 1.4.E-08 |
| NM_024409 | NPPC | 3.5 | 6.8.E-03 |
| NM_002089 | CXCL2 | 3.5 | 8.3.E-13 |
| NM_133369 | UNC5A | 3.4 | 3.2.E-02 |
| NM_198501 | SMTNL2 | 3.4 | 3.8.E-02 |
| NM_001201457 | TEX14 | 3.4 | 2.3.E-04 |
| NM_030761 | WNT4 | 3.4 | 3.2.E-02 |
| NM_152588 | TMTC2 | 3.4 | 8.1.E-03 |

**Table S4. Top 50 significant downregulated genes in LPS+JQ1-treated HMC3 cells.**

| **Gene Accession_ID** | **Gene Symbol** | **log_2_FoldChange** | ***p*-value** |
| --- | --- | --- | --- |
| NM_005328 | HAS2 | -5.5 | 2.3.E-54 |
| NM_022147 | RTP4 | -5.3 | 6.0.E-05 |
| NM_001003927 | EVI2A | -5.2 | 7.6.E-05 |
| NM_001137674 | ZNF860 | -4.6 | 4.0.E-03 |
| NM_001037735 | ZNF630 | -4.5 | 1.3.E-03 |
| NM_023915 | GPR87 | -4.4 | 5.8.E-03 |
| NM_001195483 | SLC12A8 | -4.3 | 1.1.E-02 |
| NM_001278094 | GDNF-AS1 | -4.2 | 9.8.E-03 |
| NM_001080156 | ARHGAP9 | -4.2 | 1.2.E-02 |
| NM_001199642 | ADCY5 | -4.1 | 1.5.E-02 |
| NM_004751 | GCNT3 | -4.1 | 4.3.E-03 |
| NM_001039792 | HRCT1 | -4.1 | 5.4.E-03 |
| NM_001318810 | SLITRK3 | -4.0 | 2.1.E-02 |
| NR_037803 | BACE1-AS | -4.0 | 2.3.E-02 |
| NR_109815 | FAM83C-AS1 | -3.9 | 9.0.E-03 |
| NR_038913 | NAGPA-AS1 | -3.9 | 2.8.E-02 |
| NM_024988 | CEBPA-AS1 | -3.8 | 3.2.E-02 |
| NM_001322457 | KRTAP4-8 | -3.8 | 1.1.E-02 |
| NM_004245 | TGM5 | -3.8 | 3.7.E-02 |
| NM_031471 | FERMT3 | -3.8 | 4.2.E-02 |
| NM_014033 | METTL7A | -3.8 | 1.3.E-02 |
| NM_001242773 | TMEM139 | -3.7 | 1.5.E-02 |
| NM_178816 | CASC2 | -3.7 | 1.6.E-02 |
| NM_000350 | ABCA4 | -3.7 | 1.7.E-02 |
| NM_001190787 | MCIDAS | -3.7 | 1.6.E-02 |
| NM_198404 | KCTD4 | -3.6 | 1.9.E-11 |
| NM_001145652 | C6orf141 | -3.6 | 5.5.E-02 |
| NM_001127364 | FAM221A | -3.6 | 2.1.E-02 |
| NM_001882 | CRHBP | -3.4 | 2.9.E-02 |
| NM_152466 | CRHR1-IT1 | -3.4 | 2.0.E-04 |
| NM_006439 | MAB21L2 | -3.4 | 3.8.E-02 |
| NR_033870 | TEX41 | -3.3 | 1.6.E-03 |
| NM_001002848 | LY6G5C | -3.3 | 3.6.E-02 |
| NR_003689 | SNORD123 | -3.3 | 4.1.E-02 |
| NM_020998 | MST1 | -3.3 | 4.2.E-02 |
| NR_110801 | LOC100507002 | -3.3 | 4.7.E-04 |
| NR_037167 | LOC730102 | -3.2 | 5.1.E-02 |
| NM_001256876 | KANK1 | -3.2 | 2.8.E-05 |
| NM_030916 | PVRL4 | -3.2 | 1.4.E-04 |
| NM_005266 | GJA5 | -3.1 | 4.0.E-03 |
| NR_130921 | LOC102724434 | -3.1 | 5.8.E-07 |
| NM_004982 | KCNJ8 | -3.1 | 1.6.E-08 |
| NM_207467 | C1orf220 | -3.0 | 2.0.E-03 |
| NM_018050 | MANSC1 | -3.0 | 7.5.E-03 |
| NR_120503 | KCNQ5-IT1 | -3.0 | 3.0.E-03 |
| NM_012135 | FAM50B | -3.0 | 7.1.E-03 |
| NM_001011720 | XKR9 | -3.0 | 3.4.E-02 |
| NM_001165252 | KRTAP2-3 | -2.9 | 4.8.E-09 |
| NM_001297650 | CD163L1 | -2.9 | 3.2.E-02 |
| NM_001037175 | SUSD4 | -2.9 | 9.2.E-04 |

**Table S5. Top 50 significant upregulated genes in JQ1-treated HMC3 cells.**

| Gene Accession_ID | Gene Symbol | log_2_FoldChange | *p*-value |
| --- | --- | --- | --- |
| NM_032420 | PCDH1 | 5.7 | 3.7.E-05 |
| NM_001145014 | RFPL4A | 5.4 | 1.3.E-04 |
| NM_033119 | NKD1 | 5.1 | 5.9.E-04 |
| NM_001289993 | ALS2CR12 | 4.9 | 1.2.E-03 |
| NM_017709 | FAM46C | 4.9 | 2.3.E-29 |
| NM_020163 | SEMA3G | 4.7 | 5.7.E-04 |
| NM_139170 | C16orf71 | 4.4 | 2.0.E-03 |
| NC_000001.11 | PTCHD2 | 4.3 | 3.5.E-04 |
| NM_014971 | EFR3B | 4.3 | 3.5.E-20 |
| NC_000007.14 | MIR593 | 4.3 | 1.1.E-02 |
| NM_001286688 | ABLIM2 | 4.3 | 1.1.E-02 |
| NM_004155 | SERPINB9 | 4.3 | 1.2.E-02 |
| NM_003378 | VGF | 4.3 | 1.2.E-02 |
| NM_001199097 | BAIAP3 | 4.3 | 1.7.E-09 |
| NC_000020.11 | MIR645 | 4.1 | 1.7.E-02 |
| NM_031309 | SCRT1 | 4.1 | 1.8.E-02 |
| NM_031475 | ESPN | 4.0 | 7.9.E-03 |
| NC_000003.12 | MIR425 | 4.0 | 8.4.E-03 |
| NM_001143957 | GPR63 | 4.0 | 2.9.E-02 |
| NM_001297562 | TNFSF4 | 3.9 | 2.8.E-02 |
| NM_006456 | ST6GALNAC2 | 3.9 | 2.8.E-02 |
| NM_144665 | SESN3 | 3.9 | 2.4.E-14 |
| NM_016642 | SPTBN5 | 3.9 | 2.9.E-02 |
| NM_032119 | ADGRV1 | 3.9 | 2.9.E-02 |
| NM_198485 | TPRG1 | 3.9 | 3.1.E-02 |
| NM_019074 | DLL4 | 3.9 | 7.8.E-11 |
| NM_133369 | UNC5A | 3.7 | 1.6.E-02 |
| NM_001271620 | ZNF423 | 3.7 | 5.0.E-02 |
| NM_001001788 | RAET1G | 3.7 | 4.8.E-02 |
| NM_001201457 | TEX14 | 3.5 | 1.2.E-04 |
| NM_001244889 | UNC5B | 3.5 | 2.8.E-06 |
| NM_001029954 | CDNF | 3.5 | 3.1.E-02 |
| NM_001321325 | KRTCAP3 | 3.4 | 1.3.E-02 |
| NM_014400 | LYPD3 | 3.4 | 4.5.E-02 |
| NM_005252 | FOS | 3.3 | 3.5.E-12 |
| NM_001009565 | CDKL4 | 3.3 | 4.4.E-02 |
| NM_181711 | GRASP | 3.3 | 1.6.E-13 |
| NM_001320321 | TMTC2 | 3.3 | 1.5.E-02 |
| NM_201589 | MAFA | 3.3 | 2.5.E-07 |
| NM_016084 | RASD1 | 3.2 | 7.9.E-06 |
| NM_024409 | NPPC | 3.2 | 1.5.E-02 |
| NM_182527 | CABP7 | 3.2 | 1.8.E-04 |
| NM_001126060 | NOS1AP | 3.2 | 4.2.E-03 |
| NM_005508 | CCR4 | 3.1 | 1.8.E-02 |
| NM_032829 | FAM222A | 3.1 | 2.3.E-30 |
| NC_000022.11 | NUP50-AS1 | 3.1 | 5.1.E-04 |
| NM_003862 | FGF18 | 3.1 | 3.8.E-05 |
| NM_005323 | HIST1H1T | 3.1 | 1.4.E-03 |
| NM_138345 | VWA5B2 | 3.0 | 1.6.E-03 |
| NM_001318950 | SLC30A3 | 3.0 | 1.2.E-07 |

**Table S6. Top 50 significant downregulated genes in JQ1-treated HMC3 cells.**

| **Gene Accession_ID** | **Gene Symbol** | **log_2_FoldChange** | ***p*-value** |
| --- | --- | --- | --- |
| NM_032587 | CARD6 | -5.5 | 7.5.E-05 |
| NM_005328 | HAS2 | -5.2 | 9.1.E-51 |
| NM_000576 | IL1B | -5.0 | 1.3.E-04 |
| NM_001039792 | HRCT1 | -4.8 | 1.2.E-03 |
| NR_110386 | LOC101927476 | -4.6 | 2.5.E-03 |
| NM_015660 | GIMAP2 | -4.4 | 1.3.E-03 |
| NM_023915 | GPR87 | -4.2 | 9.0.E-03 |
| NM_001882 | CRHBP | -4.2 | 1.0.E-02 |
| NR_003689 | SNORD123 | -4.1 | 1.6.E-02 |
| NM_152694 | ZCCHC5 | -4.0 | 4.6.E-03 |
| NR_110635 | LINC00687 | -4.0 | 1.7.E-02 |
| NM_001037175 | SUSD4 | -4.0 | 7.4.E-04 |
| NR_024476 | PAXIP1-AS2 | -4.0 | 1.8.E-02 |
| NM_001170553 | VSIG1 | -3.9 | 2.2.E-02 |
| NM_001308229 | C3orf33 | -3.9 | 7.8.E-03 |
| NR_030279 | MIR553 | -3.8 | 2.6.E-02 |
| NR_102369 | SNORD124 | -3.8 | 3.0.E-02 |
| NM_000426 | LAMA2 | -3.8 | 1.0.E-02 |
| NM_001330751 | PPARGC1A | -3.7 | 3.9.E-02 |
| NR_027118 | INHBA-AS1 | -3.7 | 4.0.E-02 |
| NM_001011720 | XKR9 | -3.6 | 1.8.E-02 |
| NM_001098518 | ADGRF5 | -3.6 | 4.9.E-02 |
| NM_001326475 | C3AR1 | -3.6 | 4.5.E-02 |
| NM_000370 | TTPA | -3.6 | 1.7.E-02 |
| NM_001242885 | LOC100287036 | -3.6 | 4.8.E-02 |
| NM_032037 | TSSK6 | -3.6 | 1.8.E-02 |
| NR_103450 | SACS-AS1 | -3.6 | 5.4.E-02 |
| NM_001565 | CXCL10 | -3.6 | 3.9.E-03 |
| NR_109950 | LINC01271 | -3.5 | 5.3.E-02 |
| NM_207382 | UBE2Q2P1 | -3.5 | 2.4.E-02 |
| NM_001286445 | FAM65B | -3.5 | 2.4.E-02 |
| NM_001048221 | DBNDD2 | -3.4 | 3.0.E-02 |
| NM_198404 | KCTD4 | -3.4 | 2.5.E-10 |
| NM_018295 | TMEM140 | -3.4 | 1.2.E-03 |
| NR_034144 | LINC00670 | -3.3 | 4.4.E-02 |
| NM_001330561 | HNF4G | -3.3 | 3.9.E-02 |
| NM_001013717 | C5orf56 | -3.3 | 8.8.E-03 |
| NM_005584 | MAB21L1 | -3.2 | 4.0.E-02 |
| NM_032047 | B3GNT5 | -3.2 | 5.3.E-02 |
| NM_001284308 | ADAP1 | -3.2 | 4.2.E-02 |
| NM_022147 | RTP4 | -3.2 | 5.9.E-04 |
| NR_034138 | EPHA5-AS1 | -3.1 | 5.0.E-02 |
| NM_001004317 | LIN28B | -3.1 | 5.3.E-02 |
| NM_001168319 | EDN1 | -3.0 | 2.0.E-26 |
| NM_014271 | IL1RAPL1 | -3.0 | 4.9.E-05 |
| NM_001271816 | DCLRE1A | -3.0 | 3.9.E-20 |
| NM_005266 | GJA5 | -2.9 | 7.4.E-03 |
| NM_153838 | ADGRF4 | -2.9 | 2.9.E-02 |
| NM_000584 | CXCL8 | -2.8 | 2.6.E-15 |
| NM_178550 | C1orf110 | -2.8 | 1.1.E-02 |

**Table S7. The DEGs of inflammatory genes.**

| **Gene Symbol** | **JQ1** | | **LPS** | | | **LPS+JQ1** | | |
| --- | --- | --- | --- | --- | --- | --- | --- | --- |
|  | **log_2_**  **Fold**  **Change** | ***p*-value** | | **log_2_**  **Fold**  **Change** | ***p*-value** | | **log_2_**  **Fold**  **Change** | ***p*-value** |
| CCL20 | -0.7 | 8.6.E-01 | | 6.5 | 1.6E-07 | | 8.7 | 2.7E-13 |
| CSF3 | NA | NA | | 5.6 | 5.1E-05 | | 5.5 | 7.2E-05 |
| LTB | 1.2 | 7.6.E-01 | | 5.0 | 5.6E-04 | | 1.9 | 5.5E-01 |
| CXCL10 | -3.6 | 3.9.E-03 | | 5.0 | 2.7E-59 | | 1.8 | 6.6E-05 |
| TNF | NA | NA | | 4.7 | 1.8E-03 | | 5.7 | 4.7E-05 |
| CXCL8 | -2.8 | 2.6.E-15 | | 4.6 | 0.0E+00 | | 3.9 | 1.1E-226 |
| TNFSF18 | NA | NA | | 4.6 | 3.4E-03 | | NA | NA |
| CCL2 | -1.5 | 2.4.E-01 | | 4.4 | 2.8E-18 | | 1.5 | 3.4E-02 |
| CXCL11 | -0.9 | 2.9.E-01 | | 4.1 | 4.2E-33 | | 0.1 | 9.4E-01 |
| ZBP1 | -0.7 | 8.6.E-01 | | 4.0 | 6.8E-03 | | 0.1 | 9.9E-01 |
| CSF2 | -3.0 | 1.6.E-01 | | 4.0 | 1.5E-08 | | 0.8 | 5.2E-01 |
| CXCL6 | 0.0 | 9.9.E-01 | | 3.6 | 1.0E-03 | | 2.5 | 4.9E-02 |
| CXCL1 | -0.2 | 6.6.E-01 | | 3.5 | 1.1E-46 | | 4.6 | 1.1E-89 |
| IL1B | -5.0 | 1.3.E-04 | | 3.2 | 2.3E-30 | | -0.2 | 7.4E-01 |
| TNFRSF9 | -0.2 | 7.2.E-01 | | 3.2 | 1.3E-21 | | 2.4 | 2.0E-10 |
| CXCL3 | -1.0 | 4.8.E-01 | | 3.2 | 5.7E-06 | | 3.7 | 1.7E-08 |
| IFNL1 | 0.8 | 7.1.E-01 | | 3.2 | 1.7E-02 | | 2.9 | 3.5E-02 |
| GBP2 | 0.2 | 8.5.E-01 | | 3.1 | 3.9E-05 | | 1.0 | 3.3E-01 |
| IFNB1 | -2.0 | 1.9.E-01 | | 2.9 | 3.7E-06 | | 0.1 | 9.1E-01 |
| TRAF1 | -1.5 | 3.8.E-04 | | 2.8 | 7.1E-50 | | 1.1 | 2.2E-05 |
| TNFSF10 | -1.5 | 1.1.E-02 | | 2.5 | 1.1E-17 | | -2.2 | 9.6E-04 |
| IL6 | -2.2 | 3.5.E-04 | | 2.4 | 6.3E-21 | | 1.1 | 4.3E-04 |
| CXCL2 | -2.0 | 9.6.E-02 | | 2.4 | 9.8E-06 | | 3.5 | 8.3E-13 |
| CXCL5 | 0.7 | 3.6.E-01 | | 2.3 | 4.3E-06 | | 2.6 | 2.2E-07 |
| IFIT2 | -0.3 | 3.5.E-03 | | 2.3 | 2.9E-260 | | 0.1 | 2.3E-01 |
| TNFSF13B | -0.6 | 7.2.E-01 | | 2.2 | 2.0E-02 | | -0.8 | 6.5E-01 |
| TLR2 | 0.2 | 8.7.E-01 | | 2.1 | 9.0E-03 | | 1.6 | 6.7E-02 |
| SOD2 | 0.8 | 1.5.E-10 | | 2.0 | 5.1E-90 | | 2.3 | 1.6E-133 |
| CSF1 | -1.9 | 6.7.E-06 | | 1.6 | 1.8E-13 | | 0.3 | 3.8E-01 |
| IFIH1 | -1.5 | 1.2.E-07 | | 1.6 | 7.8E-24 | | -1.1 | 2.3E-05 |
| LIF | -2.6 | 2.8.E-82 | | 0.8 | 2.2E-21 | | -0.7 | 4.9E-13 |
| EDN1 | -3.0 | 2.0.E-26 | | 0.7 | 4.4E-07 | | -2.9 | 9.1E-28 |
| IL15 | -1.7 | 4.9.E-05 | | 0.7 | 1.0E-02 | | -0.7 | 2.8E-02 |
| IL7R | -2.0 | 1.3.E-76 | | 0.5 | 1.7E-12 | | -1.9 | 3.2E-78 |
| TNFRSF11B | -2.3 | 2.8.E-03 | | 0.4 | 3.3E-01 | | -1.1 | 6.9E-02 |
| TNFSF9 | 1.9 | 8.8.E-11 | | 0.4 | 2.3E-01 | | 2.0 | 3.9E-12 |
| INHBA | -2.0 | 8.0.E-24 | | 0.4 | 7.1E-04 | | -0.8 | 1.9E-07 |
| WNT5A | -1.6 | 2.9.E-24 | | 0.4 | 6.6E-05 | | -1.4 | 1.2E-19 |
| CASP8 | -1.6 | 3.0.E-08 | | 0.3 | 1.2E-01 | | -1.7 | 7.3E-09 |
| CSF2RB | -1.6 | 1.6.E-02 | | 0.2 | 6.3E-01 | | -1.5 | 1.8E-02 |
| KITLG | 1.5 | 7.3.E-10 | | 0.2 | 4.7E-01 | | 1.6 | 3.3E-12 |
| TNFRSF10D | 1.5 | 1.4.E-42 | | 0.2 | 1.4E-01 | | 1.7 | 3.8E-59 |
| TGFB2 | -1.4 | 3.8.E-21 | | 0.1 | 6.1E-01 | | -1.6 | 4.0E-26 |
| IFNLR1 | -1.5 | 6.7.E-03 | | 0.0 | 9.9E-01 | | -2.0 | 6.6E-04 |
| GDF15 | -1.8 | 3.2.E-05 | | -0.2 | 4.9E-01 | | -0.8 | 3.1E-02 |
| CLCF1 | -1.7 | 1.5.E-10 | | -0.2 | 2.3E-01 | | -1.6 | 1.6E-10 |
| BMP6 | 1.9 | 7.1.E-02 | | -0.3 | 8.6E-01 | | 2.2 | 4.3E-02 |
| TNFRSF25 | -1.1 | 1.5.E-01 | | -0.9 | 1.9E-01 | | -2.2 | 1.3E-02 |
| NGFR | 2.3 | 6.4.E-14 | | -0.9 | 8.0E-02 | | 2.4 | 8.2E-15 |

**Table S8. The DEGs of migratory genes.**

| **Gene Symbol** | **JQ1** | | **LPS** | | | | **LPS+JQ1** | |
| --- | --- | --- | --- | --- | --- | --- | --- | --- |
|  | **log_2_**  **Fold**  **Change** | ***p*-value** | | **log_2_**  **Fold**  **Change** | ***p*-value** | **log_2_**  **Fold**  **Change** | | ***p*-value** |
| CCL20 | -0.7 | 8.6.E-01 | | 6.5 | 1.6.E-07 | 8.7 | | 2.7.E-13 |
| CSF3 | NA | NA | | 5.6 | 5.1.E-05 | 5.5 | | 7.2.E-05 |
| MMP3 | NA | NA | | 5.5 | 7.2.E-05 | 2.9 | | 2.3.E-01 |
| LTB | 1.2 | 7.6.E-01 | | 5.0 | 5.6.E-04 | 1.9 | | 5.5.E-01 |
| CXCL10 | -3.6 | 3.9.E-03 | | 5.0 | 2.7.E-59 | 1.8 | | 6.6.E-05 |
| TNF | NA | NA | | 4.7 | 1.8.E-03 | 5.7 | | 4.7.E-05 |
| CXCL8 | -2.8 | 2.6.E-15 | | 4.6 | 0.0.E+00 | 3.9 | | 1.1.E-226 |
| TNFSF18 | NA | NA | | 4.6 | 3.4.E-03 | NA | | NA |
| CD69 | NA | NA | | 4.5 | 3.7.E-03 | 1.8 | | 5.9.E-01 |
| MIR146A | NA | NA | | 4.4 | 6.0.E-03 | 3.8 | | 2.5.E-01 |
| CCL2 | -1.5 | 2.4.E-01 | | 4.4 | 2.8.E-18 | 1.5 | | 3.4.E-02 |
| MMP12 | NA | NA | | 4.3 | 9.1.E-03 | NA | | NA |
| CXCL11 | -0.9 | 2.9.E-01 | | 4.1 | 4.2.E-33 | 0.1 | | 9.4.E-01 |
| CSF2 | -3.0 | 1.6.E-01 | | 4.0 | 1.5.E-08 | 0.8 | | 5.2.E-01 |
| CXCL6 | 0.0 | 9.9.E-01 | | 3.6 | 1.0.E-03 | 2.5 | | 4.9.E-02 |
| CXCL1 | -0.2 | 6.6.E-01 | | 3.5 | 1.1.E-46 | 4.6 | | 1.1.E-89 |
| IL1B | -5.0 | 1.3.E-04 | | 3.2 | 2.3.E-30 | -0.2 | | 7.4.E-01 |
| TNFRSF9 | -0.2 | 7.2.E-01 | | 3.2 | 1.3.E-21 | 2.4 | | 2.0.E-10 |
| CXCL3 | -1.0 | 4.8.E-01 | | 3.2 | 5.7.E-06 | 3.7 | | 1.7.E-08 |
| IFNL1 | 0.8 | 7.1.E-01 | | 3.2 | 1.7.E-02 | 2.9 | | 3.5.E-02 |
| C3AR1 | -3.6 | 4.5.E-02 | | 3.1 | 2.4.E-06 | -0.9 | | 4.6.E-01 |
| PHACTR1 | 0.7 | 7.2.E-01 | | 3.0 | 2.5.E-02 | 0.0 | | 9.8.E-01 |
| VCAM1 | -0.6 | 8.0.E-01 | | 2.9 | 3.4.E-02 | -0.9 | | 7.4.E-01 |
| IFNB1 | -2.0 | 1.9.E-01 | | 2.9 | 3.7.E-06 | 0.1 | | 9.1.E-01 |
| EFNA1 | 1.0 | 8.4.E-03 | | 2.7 | 8.0.E-22 | 2.8 | | 9.9.E-22 |
| PTX3 | -0.6 | 2.0.E-05 | | 2.7 | 4.3.E-225 | 2.8 | | 5.0.E-259 |
| MMP13 | 0.3 | 8.1.E-01 | | 2.6 | 1.7.E-03 | 0.3 | | 7.8.E-01 |
| SERPINB2 | -0.9 | 4.8.E-01 | | 2.6 | 9.3.E-05 | -0.8 | | 5.3.E-01 |
| TNFSF10 | -1.5 | 1.1.E-02 | | 2.5 | 1.1.E-17 | -2.2 | | 9.6.E-04 |
| IL6 | -2.2 | 3.5.E-04 | | 2.4 | 6.3.E-21 | 1.1 | | 4.3.E-04 |
| CXCL2 | -2.0 | 9.6.E-02 | | 2.4 | 9.8.E-06 | 3.5 | | 8.3.E-13 |
| CXCL5 | 0.7 | 3.6.E-01 | | 2.3 | 4.3.E-06 | 2.6 | | 2.2.E-07 |
| IFIT2 | -0.3 | 3.5.E-03 | | 2.3 | 2.9.E-260 | 0.1 | | 2.3.E-01 |
| TNFSF13B | -0.6 | 7.2.E-01 | | 2.2 | 2.0.E-02 | -0.8 | | 6.5.E-01 |
| CD34 | -0.5 | 4.1.E-01 | | 2.1 | 1.7.E-08 | -0.8 | | 2.1.E-01 |
| CTSS | 0.4 | 5.8.E-01 | | 2.1 | 1.2.E-05 | 0.2 | | 7.2.E-01 |
| TLR2 | 0.2 | 8.7.E-01 | | 2.1 | 9.0.E-03 | 1.6 | | 6.7.E-02 |
| SOD2 | 0.8 | 1.5.E-10 | | 2.0 | 5.1.E-90 | 2.3 | | 1.6.E-133 |
| TNFAIP3 | -1.7 | 3.4.E-10 | | 1.9 | 3.7.E-44 | 1.2 | | 4.2.E-14 |
| MAP3K8 | 2.0 | 1.9.E-06 | | 1.9 | 3.2.E-06 | 3.4 | | 1.6.E-22 |
| NFKBIA | -0.1 | 5.7.E-01 | | 1.9 | 1.1.E-65 | 2.5 | | 1.4.E-124 |
| CFH | -1.0 | 1.7.E-01 | | 1.8 | 1.0.E-05 | 0.7 | | 1.7.E-01 |
| ICAM1 | -2.4 | 1.8.E-01 | | 1.8 | 3.0.E-02 | 1.9 | | 2.9.E-02 |
| IL15RA | -1.8 | 2.6.E-01 | | 1.7 | 3.5.E-02 | 0.1 | | 9.2.E-01 |
| ZC3H12A | 0.6 | 2.8.E-02 | | 1.7 | 1.8.E-13 | 2.4 | | 1.4.E-31 |
| MIR193A | 1.7 | 4.8.E-02 | | 1.7 | 4.1.E-02 | 1.5 | | 8.0.E-02 |
| CSF1 | -1.9 | 6.7.E-06 | | 1.6 | 1.8.E-13 | 0.3 | | 3.8.E-01 |
| IDO1 | -0.5 | 3.7.E-01 | | 1.6 | 7.6.E-06 | -0.9 | | 8.7.E-02 |
| GBP1 | -0.7 | 2.6.E-04 | | 1.6 | 4.2.E-34 | -0.3 | | 6.9.E-02 |
| SOCS1 | 1.4 | 6.4.E-04 | | 1.6 | 3.6.E-05 | 1.5 | | 1.4.E-04 |
| CFB | -0.3 | 4.1.E-01 | | 1.5 | 6.0.E-08 | -0.1 | | 8.9.E-01 |
| CD274 | -1.3 | 6.5.E-11 | | 1.5 | 8.4.E-36 | -0.7 | | 2.8.E-04 |
| ATF3 | 1.4 | 2.6.E-03 | | 1.5 | 6.0.E-04 | 1.6 | | 1.8.E-04 |
| APOE | 0.2 | 7.5.E-01 | | -1.5 | 5.4.E-02 | 0.2 | | 7.4.E-01 |
| MUC2 | -1.2 | 4.1.E-01 | | -4.0 | 2.3.E-02 | -1.4 | | 3.2.E-01 |
